# Single-cell RNA sequencing reveals roles of unique retinal microglia types in early diabetic retinopathy

**DOI:** 10.1186/s13098-024-01282-3

**Published:** 2024-02-26

**Authors:** Yan Wang, Xiongyi Yang, Yuxi Zhang, Libing Hong, Zhuohang Xie, Wenmin Jiang, Lin Chen, Ke Xiong, Siyu Yang, Meiping Lin, Xi Guo, Qiumo Li, Xiaoqing Deng, Yanhui Lin, Mingzhe Cao, Guoguo Yi, Min Fu

**Affiliations:** 1 Department of Ophthalmology, South China Hospital, Medical School, Shenzhen University, Shenzhen, 518116 People’s Republic of China; 2 The Second Clinical School, Southern Medical University, Guangzhou, Guangdong People’s Republic of China; 3 State Key Laboratory of Organ Failure Research, National Clinical Research Center of Kidney Disease, Guangdong Provincial Institute of Nephrology, Nanfang Hospital, Southern Medical University, Guangzhou, People’s Republic of China; 4 Department of Ophthalmology, The Second Xiangya Hospital of Central South University, Changsha, 410011 Hunan People’s Republic of China; 5 Hunan Clinical Research Center of Ophthalmic Disease, Changsha, 410011 Hunan People’s Republic of China; 6 Department of Anesthesiology, Shenzhen Hospital, Southern Medical University, 1333 Xinhu Road, Shenzhen, 518100 Guangdong People’s Republic of China; 7 Department of Ophthalmology, Nanfang Hospital, Southern Medical University, Guangzhou, 510515 Guangdong People’s Republic of China; 8 Department of Ophthalmology, The Seventh Affiliated Hospital, Sun Yat-Sen University, Shenzhen, People’s Republic of China; 9 School of Rehabilitation Medicine, Southern Medical University, Guangzhou, Guangdong People’s Republic of China; 10 Health Management Center, The Third Xiangya Hospital, Central South University, Changsha, 410013 Hunan People’s Republic of China; 11 Department of Ophthalmology, The Sixth Affiliated Hospital, Sun Yat-Sen University, No. 26, Erheng Road, Yuancun, Tianhe, Guangzhou, Guangdong People’s Republic of China; 12 Biomedical Innovation Center, The Sixth Affiliated Hospital, Sun Yat-Sen University, Guangzhou, People’s Republic of China; 13 Department of Ophthalmology, Zhujiang Hospital, Southern Medical University, Guangzhou, Guangdong People’s Republic of China

**Keywords:** Diabetic retinopathy, Retinal microglia, Macrophages, Inflammation, Single-cell RNA sequencing

## Abstract

**Background:**

The pathophysiological mechanisms of diabetic retinopathy (DR), a blinding disease, are intricate. DR was thought to be a microvascular disease previously. However, growing studies have indicated that the retinal microglia-induced inflammation precedes microangiopathy. The binary concept of microglial M1/M2 polarization paradigms during inflammatory activation has been debated. In this study, we confirmed microglia had the most significant changes in early DR using single-cell RNA sequencing.

**Methods:**

A total of five retinal specimens were collected from donor SD rats. Changes in various cells of the retina at the early stage of DR were analyzed using single-cell sequencing technology.

**Results:**

We defined three new microglial subtypes at cellular level, including two M1 types (*Egr2*^+^ M1 and *Egr2*^−^ M1) and one M2 type. We also revealed the anatomical location between these subtypes, the dynamic changes of polarization phenotypes, and the possible activation sequence and mutual activation regulatory mechanism of different cells. Furthermore, we constructed an inflammatory network involving microglia, blood-derived macrophages and other retinal nonneuronal cells. The targeted study of new disease-specific microglial subtypes can shorten the time for drug screening and clinical application, which provided insight for the early control and reversal of DR.

**Conclusions:**

We found that microglia show the most obvious differential expression changes in early DR and reveal the changes in microglia in a high-glucose microenvironment at the single-cell level. Our comprehensive analysis will help achieve early reversal and control the occurrence and progression of DR.

**Supplementary Information:**

The online version contains supplementary material available at 10.1186/s13098-024-01282-3.

## Introduction

The pathophysiological mechanisms of diabetic retinopathy (DR), a blinding disease, are intricate [1]. The retina is composed of 11 cell types, including neuronal cells, glial cells and vascular bed cells, the interaction of which affects the homeostasis of retina. DR was thought to be a microvascular disease previously. However, growing studies have indicated that the retinal microglia-induced inflammation precedes microangiopathy [2]. Currently, DR is diagnosed and treated according to microvascular manifestations in retinal image examinations, such as microaneurysms and retinal hemorrhage, which lag far behind the actual pathological changes [3]; this results in irreversible retinal nerve tissue damage and visual impairment [4]. Therefore, it is important to prevent blindness in patients by identifying early DR more precisely based on understanding of the pathological and molecular mechanisms in retinal microglia-induced inflammation.

Microglia, a type of mononuclear phagocytic cells, are regarded as resident macrophages in retina and brain. Microglia can be classified into M1 type (pro-inflammatory state) and M2 type (anti-inflammatory state) according to their activated phenotypic characteristics [5]. M1-type microglia are neurotoxic, while M2-type microglia are neuroprotective. The M1/M2 classification was originally used to classify the different activation states of macrophages [6]. Interestingly, the binary concept of microglial M1/M2 polarization paradigms has recently been debated. In vivo studies demonstrate the intermediate phenotype in microglia, suggesting that the distinction between M1 and M2 microglia is ambiguous and that microglia can adjust their phenotype according to disease progression [7]. It was suggested that microglia presented a continuous and adjustable spectrum of phenotypes [8, 9]. Today, obviously the M1/M2 classification oversimplifies the real complexity of microglia, which is detrimental to the study of DR. Hence, studying the activation phenotype of microglia can better explain the disease pathogenesis.

Little is known about the activation patterns and phenotypic changes of microglia during retinal disease. Activation of microglia is considered a hallmark of neuroinflammation, with typical morphological changes and expression of surface markers during disease progression. The phenotypic, morphological, and functional characteristics of retinal microglia may be highly correlated with the signaling events that trigger their activation. After being activated, the morphology of microglia changed from branching to amoeba-like, accompanied by the migration of microglia and the release of a large number of inflammatory factors [10]. As an effective method to study cellular heterogeneity, single-cell RNA sequencing (scRNA-seq) can discover rare cell types, accurately reflect the gene expression of a single cell, and reveal the differential expression between cells. It has been widely used in the exploration of disease mechanism [11, 12]. O'Koren et al. used scRNA-seq to reveal the unique transcriptome-related genes of microglia in photoreceptor degeneration, and differentiated microglia into two subtypes, which both related to pigment epithelium protection [13].

Herein, by means of scRNA-seq of retinal tissues of normal rats and diabetic rats at different weeks, we constructed a transcriptional map of the rat retina for the first time and discovered new disease-specific microglia subtypes. We revealed the changes of microglia in DR and their relationship with other retinal cell types. Furthermore, we obtained evidence that activated retinal microglia may exhibit a continuous phenotypic spectrum, which is significant for studying the effect of microglia in the intermediate state between M1/M2 on DR. Our comprehensive analysis provides disruptive meaning and new targets for prediction, diagnosis and treatment of DR.

## Materials and methods

### Animals

A total of five retinal specimens were collected from donor SD rats, including two at 0 weeks (control group) and one each after 2 weeks, 4 weeks, and 8 weeks of DR (experimental group). The SD rats were acclimatized for 1 week on a standard diet. After acclimatization, diabetes was induced in the experimental rats with STZ after overnight fasting (Sigma, St. Louis, MO, USA; 45 mg/kg, IP). The STZ was freshly dissolved in citrate buffer (0.1 M) and maintained on ice before use. Two randomly selected rats, referred to as the control rats, were injected intraperitoneally with 10 mL of 0.1 M citrate phosphate buffer per kg of body weight after 12 h of fasting. The remaining rats, referred to as the experimental rats, were intraperitoneally administered 45 mg of STZ per kg of body weight in a single injection. Induction of diabetes was confirmed by the fasting blood glucose concentration on the third day after administration of STZ. Rats with blood glucose levels above 200 mg/dL were considered to have diabetes and were used for the experiment. Animal-specific blood glucose levels and body weight changes can be seen in the Additional file 1: Table S3.

### Tissue processing and cell purification

Animals that were considered diabetic were maintained on a high-sugar diet, and tail vein blood samples were collected and measured regularly for assessment of glucose levels. The rats in the experimental group (2 weeks, 4 weeks, 8 weeks) and the control group were anesthetized by intraperitoneal injection and fixed in the supine position. First, the eyeballs were removed, and then the corneoscleral limbus was cut. The eyeballs were radially incised, the anterior segment and vitreous were removed.

The tissues were transported in sterile culture dishes with 10 ml of 1 × Dulbecco's phosphate-buffered saline (DPBS; Thermo Fisher, Cat. No. 14190144) on ice. The residual tissue storage solution was removed, and the tissues were then minced on ice. We used 0.25% trypsin (dissociation enzyme; Thermo Fisher, Cat. No. 25200-072) and 10 µg/mL DNase I (Sigma, Cat. no. 11284932001) dissolved in PBS with 5% fetal bovine serum (FBS; Thermo Fisher, Cat. No. SV30087.02) to digest the tissues. The tissues were dissociated at 37 °C with a shaking speed of 50 RPM for approximately 40 min. We repeatedly collected the dissociated cells at intervals of 20 min to increase cell yield and viability. The cell suspensions were filtered using a 40 µm nylon cell strainer, and red blood cells were removed by 1X Red Blood Cell Lysis Solution (Thermo Fisher, Cat. No. 00-4333-57). The dissociated cells were washed with 1 × DPBS containing 2% FBS. The cells were stained with 0.4% Trypan Blue (Thermo Fisher, Cat. No. 14190144), and viability was assessed on a Countess^®^ II Automated Cell Counter (Thermo Fisher).

### 10 × library preparation and sequencing

Beads with unique molecular identifiers (UMIs) and cell barcodes were loaded close to saturation so that each cell was paired with a bead in gel beads-in-emulsion (GEMs). After exposure to cell lysis buffer, polyadenylated RNA molecules hybridized to the beads. The beads were retrieved into a single tube for reverse transcription. In cDNA synthesis, each cDNA molecule was tagged on the 5’ end (that is, the 3’ end of the messenger RNA transcript) with the UMI and a cell label indicating its cell of origin. Briefly, 10 × beads were subjected to second-strand cDNA synthesis, adaptor ligation, and universal amplification. Sequencing libraries were prepared using randomly interrupted whole-transcriptome amplification products to enrich the 3’ ends of the transcripts linked with the cell barcodes and UMIs. All the remaining procedures, including library construction, were performed according to the standard manufacturer’s protocol (CG000206 Rev D). The sequencing libraries were quantified using a High Sensitivity DNA Chip (Agilent) on a Bioanalyzer 2100 and with a Qubit High Sensitivity DNA Assay (Thermo Fisher Scientific). The libraries were sequenced on a NovaSeq6000 instrument (Illumina) in 2 × 150 bp mode.

### scRNA-seq analysis

The data from all samples were combined in R (4.0.2) using the Read10X() function from the Seurat package (3.2.2) and generated an aggregate Seurat object. Low-quality cells (< 350 genes/cell, > 3000 genes/cell, < 3 cells/gene, > 20% mitochondrial genes and > 20% ribosomal genes) were excluded. Finally, 35910 single cells, including 11073 normal tissue-derived cells and 24837 diabetic tissue-derived cells, were subjected to further investigation. To identify cell clusters, principal component (PC) analysis (PCA) was first performed on the list of highly variable genes. Significant PCs were determined using JackStraw analysis, and the top 20 PCs were used in this process. We used the FindClusters() function to perform clustering (resolution 1.0). We use two data dimensionality reduction algorithms (2D UMAP and tSNE) for visualization. For normalized gene expression data, we used the FindAllMarkers function to list the markers of each cell cluster. Compared with that in other cells, the expression of these marker genes was upregulated by at least 1.3 times. The main cell types were identified based on markers described in the literature. One cluster that was identified as red blood cells (cluster 32, cell number 52) was removed, and a total of 10 cell types were finally obtained.

### Gene enrichment analysis

The FindMarkers function was used to identify DEGs between two clusters (adjusted p value < 0.01 and fold change [FC] > 1.3). The R package clusterProfiler was used to perform GO and Kyoto Encyclopedia of Genes and Genomes (KEGG) pathway enrichment for the DEGs. Based on the gene set data for *Rattus norvegicus* in the msigdbr package (selections: C2 for category and CP: KEGG for subcategory), we used GSEA to identify gene sets that were significantly enriched in specific cell clusters. We limited the threshold to a false discovery rate (FDR) q < 0.25 and a p < 0.1.

GSVA was performed using the same gene set as GSEA to analyze the enriched gene sets between different cell subtypes. Subsequently, the limma package was used to determine the gene sets with significant differences. Differentially enriched signatures were defined as having FDR adjusted p < 0.05 and |mean score difference| values ≥ 0.1 as described previously. Then, the pheatmap() function in the package pheatmap(1.0.12) was used to render the results as heatmaps.

### Pseudotime analysis

To understand the transition between microglial cells, trajectory analysis was conducted using Monocle2. The analysis used the newCellDataSet() function to create a monocle object from clustering analysis. After reducing the dimensionality of the data through the reduceDimension() function, the cells were ordered into pseudotime. The function plot_cell_trajectory() was used to generate plots of pseudotime by state, cluster and pseudotime. Furthermore, we analyzed different genes related to pseudotime analyses through the differentialGeneTest() function. The heatmap of clusters along the pseudotime trajectory was also generated using the plot_pseudotime_heatmap() function.

According to the results of Monocle2 analysis, the genes were divided into 5 patterns, and each pattern was then subjected to GO and KEGG enrichment analyses.

Using GeneSwitches, we first performed binarization analysis on genes in the differentiation trajectory and screened out potential switch genes with on and off states in their expression characteristics. Then, we performed logistic regression analysis and pseudotime correlation analysis on these potential switch genes to obtain the R value of each correlation. A higher pseudochronological correlation was associated with a closer relationship between the gene and the trajectory process. After obtaining the switch time and the correlation R value of each potential switch gene, the top switch genes were sorted and visualized in pseudochronological order according to their switch times. We also used GeneSwitches to obtain the enrichment analysis results for switch genes through gene annotation (such as surface proteins, transcription factors and other functional types) and enrichment analysis (GO:BP) methods.

### Cell–cell interaction analysis

To visualize and analyze intercellular communications from scRNA-seq data, we conducted CellChat analysis. We created a new CellChat object from our Seurat object. The cell types were added to the CellChat object as cell metadata. CellChat identified differentially overexpressed ligands and receptors for each cell group and associated each interaction with a probability value to quantify communications between the two cell groups mediated by these signaling genes. Significant interactions were identified on the basis of a statistical test that randomly permuted the group labels of cells and then recalculated the interaction probability. The results were visualized using a hierarchy plot, a Circos plot and a chord diagram through the netVisual_aggregate() and netVisual_heatmap() functions. Furthermore, we identified and visualized outgoing and incoming communication patterns of cells using the identifyCommunicationPatterns() and netAnalysis_river() functions with the nPatterns set as 3 and 4.

### Immunostaining

Rats were deeply anesthetized with ketamine and xylazine and then sacrificed by cervical dislocation. The eyes were immediately removed, the corneas were incised, and each eye was immersed in 2% paraformaldehyde (PFA) for 3 h at room temperature. After immersion and fixation, the eyes were washed with phosphate-buffered saline (PBS), and the lenses were carefully removed. The eyes were immersed in 10% sucrose for 30 min, 20% sucrose for 2 h, and 30% sucrose overnight at 4 °C for cryoprotection before being embedded in optimal cutting temperature (OCT) compound Tissue-Tek) and frozen on dry ice. Eighteen-micron sections were cut on a cryostat. The sections were blocked for 1 h with 10% normal goat or donkey serum in PBS with 0.5% Triton X-100, incubated overnight at 4 °C with primary antibodies, and then incubated with secondary antibodies.

### Antibody

Antibodies used in this study were as follows: Purified mouse Complement Component C1qA Antibody (NOVUS NBP1-51,139); Rabbit Monoclonal EGR2 Antibody (JG78-39); Rat monoclonal [HEK/1/85a] to CCR5 (Abcam ab11464); Cy3–conjugated Affinipure Goat Anti-Mouse IgG(H + L) (SA00009-1); Goat Anti-Rabbit IgG(H + L), Mouse/Human ads-APC (SBA-4050-11S).

### Protein–protein interaction analysis

The interactions of various proteins were obtained from the STRING database (https://cn.string-db.org/) in the form of.tsv files. These files were then visualized using the Cytoscape tool (version 3.8.2). The.tsv file of each protein was uploaded to the Cytoscape tool, and the interactions were merged manually to analyze the overall interactions. The interaction of each protein, the overall interactions of the proteins and their corresponding interactions were illustrated using Cytoscape version 3.8.2.

### Cell sorting

Experimental Methods: (1) Rat execution and eyeball removal: SD rats were killed by neck-breaking and soaked in 75% ethanol for 30 min, and the eyeballs were removed aseptically and placed in PBS. (2) Acquisition of retinal tissues: Cornea, lens and vitreous body were sequentially removed using a microscope operating on ice, retinal neuroepithelium was bluntly detached, retinal blood vessels were clipped by microscopic manipulation (on ice), and rinsed 5 times in PBS solution. (3) Preparation of retinal single-cell suspension: the above prepared tissue was placed in the prepared 1 g/L trypsin digestion solution, digested at 37 °C for 3–5 min, and then observed under the microscope to see whether it reached the single-cell state; after reaching the single-cell state under the microscope, the digestion was terminated by the addition of complete medium, centrifuged at 200 g for 10 min, and the supernatant was discarded. Add 10 mL of DMEM medium containing 0.05% BSA to resuspend the cells.

Grouping: External staining: A. FITC-CCR5; B. without FITC-CCR5, Cy3-C1qA, and APC-Egr2. Internal staining: A. Cy3-C1qA; B. APC-Egr2; C. Cy3-C1qA and APC-Egr2.

External staining: (1) Preparation of single cell suspension: cells were collected in centrifuge tubes after recovery, centrifuged (2000 rpm, 5 min) to remove residual medium, and washed twice by centrifugation with 1*PBS; cells were counted; (2) (1*10^6^) 100 μL 1*PBS resuspended precipitate was made into single cell suspension and transferred into 1.5 mL centrifuge tube; (3) Add 1 μL of FITC-CCR5 antibody, and avoid light for 1 h at 4 °C; (4) Add PBS to 2 mL, 2000 rpm, 5 min; (5) Discard the supernatant and resuspend 2 mL of PBS at 2000 rpm for 5 min.

Internal staining: (1) Centrifuge the precipitated cells and remove the supernatant, resuspend the cells at a density of about 100 μL of fixative for 1*10^6^ cells, and fix them at room temperature for about 20 min; (shaking, adhering to the wall, and rotating the drop); (2) Centrifugation (2000 rpm, 5 min) to remove fixative; (3) 2 mL 1 × permeabilizer resuspension, 2000 rpm,5 min, two times; (4) Resuspend cells at a density of approximately 100 μL of permeabilizer for 1*10^6^ cells and let stand for 10 min; (5) In each 100 μL of cell resuspension, incubate dual-labeled antibodies (C1QA and EGR2) for 1 h at 4 °C away from light, 2 μL of each; (6) Centrifuge the precipitated cells and discard the supernatant, and resuspend in 2 mL PBS, 2000 rpm, 5 min, twice; (7) Add 200 μL of diluted fluorescent secondary antibody to resuspend the cells, Cy3-1 μL, APC-0.5 μL (permeabilizer) and incubate at 4 °C for 1 h away from light; (8) Wash, centrifuge the precipitated cells, discard the supernatant, resuspend the cells with 200 μL PBS, and analyze them on a flow cytometer.

### QPCR

5 DM 4 weeks and 2 normal rats were used for the experiment. The sampling method was as described previously. The extracted total RNA was reverse transcribed according to the reverse transcription kit instructions, cDNA was synthesized, and PCR experiments were performed. After sorting out *C1qa*^+^*Ccr5*^+^*Egr2*^+^ microglia using flow cytometry, total RNA was extracted for qPCR experiments. The forward primer for the target gene *Tnf-α* is 5' TGGCGTGTTCATCCGTTCTCTACC 3' and the reverse primer is 5' CCCGCAATCCAGGCCACTACTT 3'; the forward primer for the target gene *c-Jun* is 5' CCTTCTACGACGTGCCCTCA 3' and the reverse primer is 5' GGGGTCGGTGTAGTGGTGATGT 3'; the forward primer for the target gene *Jund* is 5' ATGCTGAAGAAAGACGCGCTG 3' and the reverse primer is 5' CGCCACCCGCGAAAACTGCTCA 3'; the forward primer for the target gene *Ccl3* is 5' CCACTGCCCTTGCTGTTCTT 3' and the reverse primer is 5' GCAAAGGCTGCTGGTTTCAA 3'.

## Results

### An atlas of retinal cell types in different diabetic stages

**Fig. 1:**
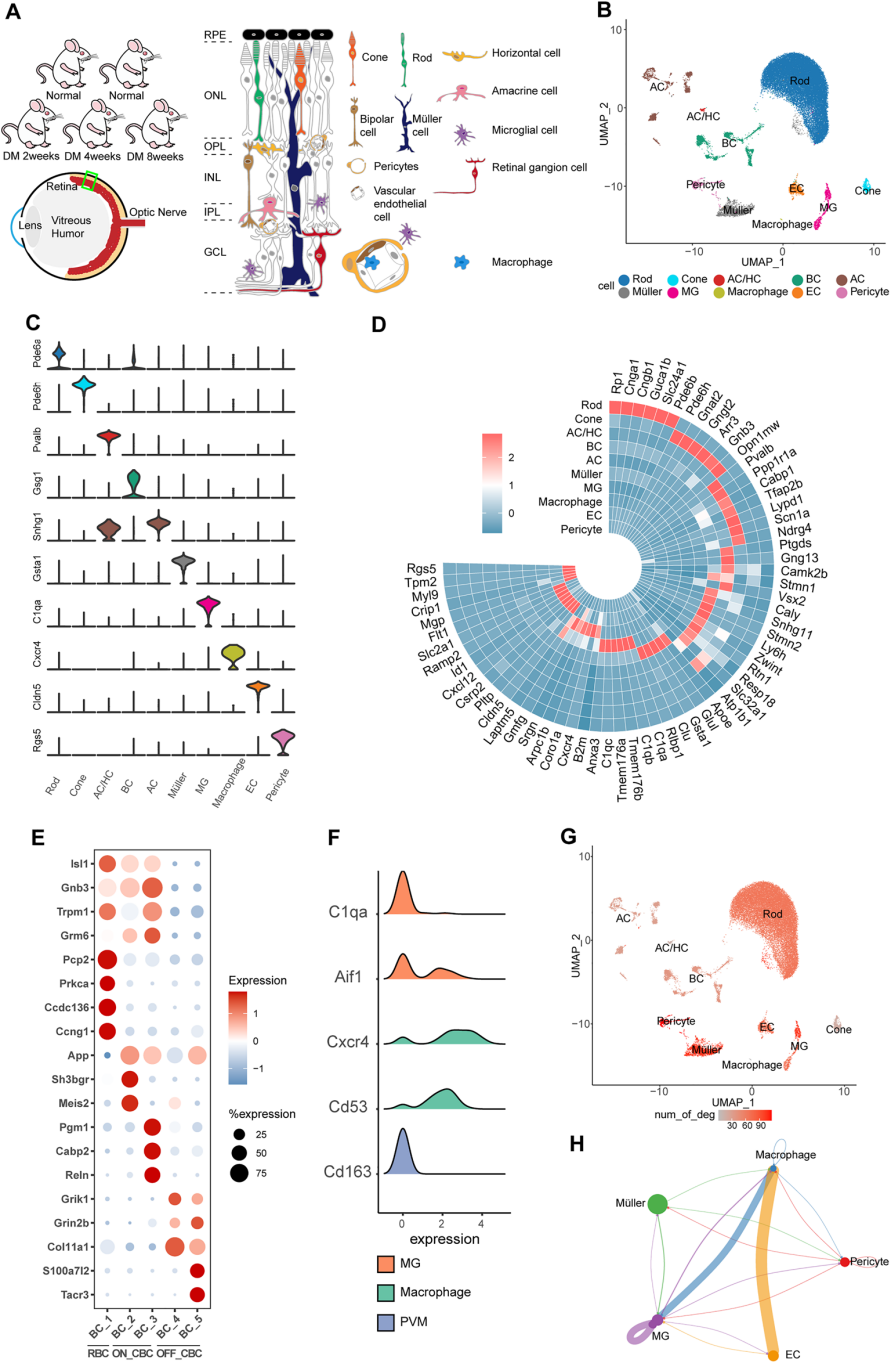
Atlas of retinal cell types in different diabetic stages. **A** Overview of the experimental strategy. Retinal cells were isolated from 2 normal SD rats and 3 diabetic rats with 2, 4 and 8 weeks of diabetes mellitus (for a detailed isolation protocol, see the Methods section). Rods and cones convert light stimulation into chemical signals and transmit the information to bipolar cells (BCs). BCs connect visual cells and ganglion cells. In addition, there are glial cells (Müller cells, microglia), horizontal cells (HCs), amniotic cells (ACs), vascular endothelial cells and pericytes. The retina is divided into different layers from the outside to the inside: the retinal pigment epithelium (RPE), outer nuclear layer (ONL), outer plexiform layer (OPL), INL, IPL, and GCL. The BRB is composed of vascular endothelial cells, pericytes and the basement membrane, which ensures the stability of the internal environment of the eye. Blood-derived macrophages can be seen in the blood vessels. **B** UMAP plot showing different cell types. Cells assigned to the same cluster are similarly colored. *AC* amacrine cell, *HC* horizontal cell, *EC* endothelial cell, *MG* microglial cell, *BC* bipolar cell. **C** Violin plot showing the expression of marker genes in each cell type. **D** Circular heatmap showing the markers for each cell type. High expression is in red, and low expression is in blue. **E** Bubble plot showing the markers of each subcluster of BCs. The size of each circle is proportional to the percentage of the gene expression. High expression is in red, and low expression is in blue. **F** Ridge plot showing the expression of markers of microglia (*C1qa*, *Aif1*), blood-derived macrophages (*Cxcr4*, *Cd53*) and perivascular macrophages (*Cd163*) in macrophages. **G** UMAP plot showing the numbers of DEGs in the cells of normal and diabetic rats. Color scale: red, large number of DEGs; gray, small number of DEGs. **H** Cell–cell communication between nonneuronal cells in inflammation-related pathways. The size of the circle and the thickness of the line represent the strength of communication. The direction of the arrow is consistent with the direction of the signal from the sender to the receiver

To simulate early DR, we used streptozotocin (STZ) to induce a type 2 diabetes model in rats [14]. We stripped the retinas of 5 rats (2 normal rats and 3 model rats treated with STZ for 2, 4, and 8 weeks) and dissociated and diluted these samples for single-cell transcriptome sequencing (Fig. 1A). After quality control, 35910 high-quality cells were retained, including 11073 normal sample-derived cells and 24837 diabetic rat-derived cells (Additional file 1: Figure S1A). We performed unbiased clustering to cluster cells with similar gene expression profiles and found 34 clusters. One type defined as red blood cells with a very small number of cells was removed, so we finally obtained 33 cell subtypes [15]. Uniform manifold approximation and projection (UMAP) dimensionality reduction was used to visualize all cell subtypes (Additional file 1: Figure S1B). We compiled a list of genes previously reported to be expressed in retinal cell types (Additional file 1: Table S1).

According to the expression of these genes in each cluster, the 33 clusters were divided into 10 cell types, including one blood-derived macrophage type (Figs. 1F and Additional file 1: Figure S2G). and retinal resident cell types. The resident cells included five neuronal cell types (rod cells [rods], cone cells [cones], horizontal cells [HCs], amacrine cells [ACs], and bipolar cells [BCs]), two glial cell types (Müller cells and microglia), endothelial cells and pericytes (Fig. 1B, C and Additional file 1: Figure S1C-E). Apart from canonical cell-type markers, we also identified additional genes that strongly and specifically marked each major cell population (Fig. 1D and Additional file 1: Figure S1F, Table S2).

Since almost no ganglion cells were detected, we conducted an in-depth analysis of five neuronal types. In our samples, rods accounted for the vast majority (71%) of the retinal tissue. Rods mainly sense weak light [16], which is consistent with the results of our Gene Ontology (GO) enrichment analysis (Additional file 1: Figure S2A). In previous studies, cones were divided into three subtypes based on the expression of opsin genes (*Opn1sw*, *Opn1mw* and *Opn1lw*) [17]. We detected only *Opn1sw* and *Opn1mw* because the sequence homology between them is approximately 99.8% [18], so we divided cones into *Opn1sw*^+^ cones (S-cones) and *Opn1mw*^+^ cones (M-cones). There were also *Opn1sw*^+^/*Opn1mw*^+^ and *Opn1sw*^*−*^/*Opn1mw*^*−*^ cones in the retina (Additional file 1: Figures S2B, C). According to the expression of known marker genes [19, 20], we divided BCs and ACs into different subtypes (Fig. 1D, E and Additional file 1: Figure S2D, E). Specially, some markers ubiquitously expressed in BCs and markers used to distinguish BC subclasses were newly discovered (Fig. 1E and Additional file 1: Figure S2D). One cluster that contained HCs and a small number of ACs highly expressed HC markers (*Onecut1*, etc.) [21] and A-II glycinergic AC markers (*Slc6a9* and *Gjd2*) [20] (Additional file 1: Figure S2E, F).

We identified the differentially expressed genes (DEGs) between normal and diabetic rats in each cell type. The most significant changes were found in nonneuronal cells (Fig. 1G). We selected the main inflammatory pathways and analyzed the cell communication between nonneuronal cells with CellChat [22]. Microglia and macrophages dominated during inflammation, and endothelial cells were also significantly involved in inflammation in early DR (Fig. 1H and Additional file 1: Figure S2H). Therefore, we next explored the molecular mechanisms of retinal nonneuronal cells in the inflammatory regulatory network during DR.

### Inflammation and activation characteristics of microglial subtypes

**Fig. 2:**
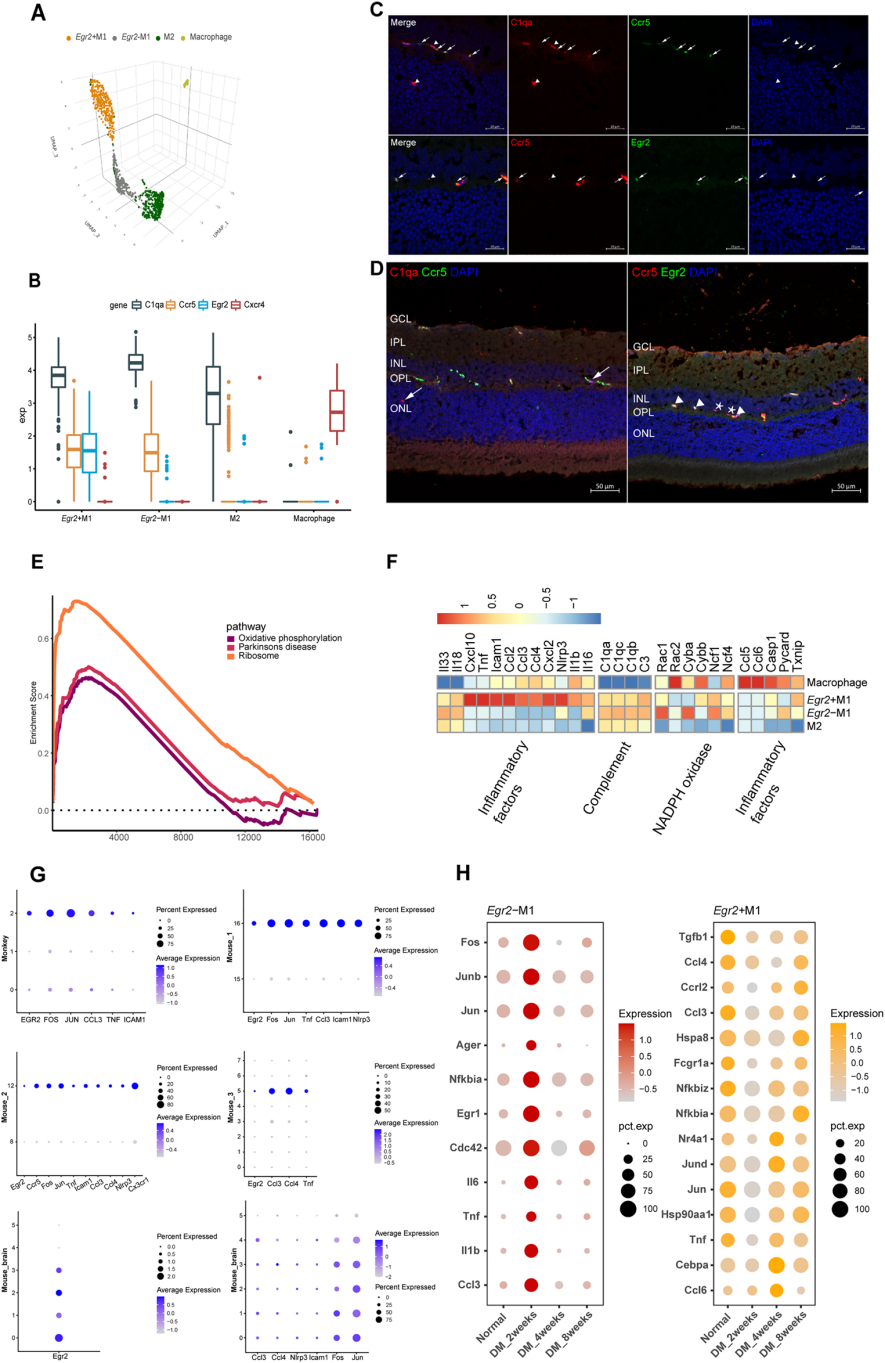
Inflammatory and activation characteristics of microglial subtypes. A Three-dimensional UMAP plot of microglia and macrophages. The different colors correspond to different cell types. **B** Boxplot showing the marker genes of microglia and macrophages. According to their different gene expression profiles, the microglia were categorized into three groups: *Egr2*^+^ M1 microglia (*C1qa*^+^, *Ccr5*^+^, *Egr2*^+^), *Egr2*^*−*^ M1 microglia (*C1qa*^+^, *Ccr5*^+^, *Egr2*^*−*^) and M2 microglia (*C1qa*^+^, *Ccr5*^*−*^, *Egr2*^*−*^). Macrophages highly expressed *Cxcr4*. **C** Immunofluorescence labeling for *C1qa* (red) and *Ccr5* (green) and DAPI nuclear staining (blue) in the rat retina. M1 microglia are indicated by arrows, and M2 microglia are indicated by arrowheads (above, 63 ×). Immunofluorescence labeling for *Ccr5* (red) and *Egr2* (green) and DAPI nuclear staining (blue) in the rat retina. *Egr2*^+^ M1 microglia are indicated by arrows, and *Egr2*^*−*^ M1 microglia are indicated by arrowheads (below, 63 ×). Scale bar 20 µm. **D** Immunofluorescence labeling for C1qa (red) and *Ccr5* (green) and DAPI nuclear staining (blue). M2 microglia are indicated by arrows (left, 20 ×). Immunofluorescence labeling for *Ccr5* (red) and *Egr2* (green) and DAPI nuclear staining (blue). *Egr2*^+^ M1 microglia are indicated by arrowheads, and *Egr2*^*−*^ M1 microglia are indicated by asterisks (right, 20 ×). Scale bar 50 µm. **E** Plot showing the pathways enriched in M2 microglia in GSEA. The threshold limits were a p value < 0.1 and a FDR q-value < 0.25. **F** Heatmap showing the average expression of inflammatory mediators in microglia and macrophages. The plot is ordered by the three gene categories (labels on left). High expression is in red, and low expression is in blue. **G** Bubble plots showing the expression of inflammatory cytokines in microglia subpopulations from different data sets. The size of each circle is proportional to the percentage of gene expression. High expression is shown in blue, and low expression is shown in gray. Mouse_2, Mouse_3 and Mouse_brain were obtained from normal mouse samples. **H** Bubble plot showing the expression of inflammatory mediators in *Egr2*^+^ M1 microglia and *Egr2*^*−*^ M1 microglia in different stages of DR. The size of each circle is proportional to the percentage of gene expression. High expression is shown in red/yellow, and low expression is shown in gray

Microglia can be activated to a proinflammatory M1-like or anti-inflammatory M2-like phenotype [23, 24], as observed in our study. Based on the expression of known microglial markers (*C1qa*) [25], we identified three microglial subtypes (Fig. 2A and Additional file 1: Figure S3A, B). In our study, *Ccr5* expression was used to distinguish M1 microglia from M2 microglia. According to the expression of *Egr2*, the M1 microglia were divided into *Egr2*^+^ M1 microglia and *Egr2*^*−*^ M1 microglia (Fig. 2B and Additional file 1: Figure S3C). We verified the existence of the three microglial types by immunofluorescence staining and identified the locations of the three subtypes (Fig. 2C and Additional file 1: Figure S3D, E). M1 microglia were located in the inner nuclear layer (INL) and M2 microglia were mainly located in the INL and ONL, with a small number located in the inner plexiform layer (IPL) (Fig. 2D and Additional file 1: Figure S3F). Collectively, these results suggest that M1 and M2 microglia have a different spatial distribution in the retina and thus may play different roles.

To reveal the different roles of these three subgroups of microglia in DR, we compared their features, specifically their inflammatory characteristics. The M2 microglia had low expression of inflammatory factors, which is consistent with the findings of previous studies [26]. We used gene set enrichment Analysis (GSEA) to assess the heterogeneity of M1 and M2 microglia [27]. In M2 microglia, oxidative phosphorylation was the main metabolic pathway, which was consisted with prior research; this pathway may provide sufficient energy in the long term [28, 29]. The ribosome pathway was also enriched in M2 microglia, suggesting that it may synthesize a large number of proteins to meet the high metabolic demands of the cells (Fig. 2E). The two M1 subgroups cause retinal damage by releasing distinct inflammatory factors. *Egr2*^+^ M1 microglia release stronger proinflammatory cytokines, such as *Tnf*, *Il1β*, *Nlrp3*, and *Icam* [30]*.* *Egr2*^*−*^ M1 microglia mediate the inflammatory response by activating the complement system and secreting weaker proinflammatory factors, such as *Il33* and *Il18* [31, 32]*.* The activation of the retinal complement system usually mediates chronic low-grade tissue inflammation, which is considered to be the main inflammatory manifestation in early DR [33–35] (Fig. 2F). Chemokines can recruit leukocytes to further aggravate inflammation [35]. In our study, *Egr2*^+^ M1 microglia strongly expressed multiple chemokines, such as *Cxcl10* and *Ccl2* (Fig. 2F). In addition, NADPH oxidase expression can be upregulated by activating Rac1, leading to the production of reactive oxygen species (ROS), substances that markedly aggravate retinal oxidative stress [36]. Interestingly, *Egr2*^*−*^ M1 microglia also strongly expressed Rac1 and NADPH oxidase-related genes (Fig. 2F). In conclusion, we found that the pro-inflammatory effect of *Egr2*^+^ M1 microglia was stronger than that of *Egr2*^*−*^ M1 microglial since *Egr2*^+^ M1 microglia participated in the regulation of inflammation in more ways and produced factors with stronger pro-inflammatory effects.

To validate our findings, we performed analyses using single-cell datasets from monkeys and multiple mice, respectively, where the microglia data from mice were derived from eye and brain tissue. Interestingly, we found one or more subpopulations of *Egr2*^+^M1-like microglia, which were also active and more inflammatory than other microglia subpopulations (Fig. 2G). This finding suggests that this cell population may play an essential role in different species and even different tissues.

Next, we analyzed these three subtypes' order of activation and cellular status during DR (Fig. 2H). *Egr2*^+^ M1 microglia downregulated inflammatory factor expression early (at 2 weeks of DR) and then upregulated inflammatory factors after 4 weeks of DR. It has been suggested that activated microglia have a "self-control" mechanism to limit their own activation and limit the damage associated with unresolved inflammation [37–39]. In this way, microglia are able not only to generate an immune response but also to suppress inflammation and restore homeostasis [40]. We speculate that *Egr2*^+^ M1 microglia may suppress autoinflammation in response to a hyperglycemic environment early in the DR, preventing premature activation and overproduction of proinflammatory cytokines. Compared with *Egr2*^+^ M1 microglia, *Egr2*^*−*^ M1 microglia exhibited upregulation of inflammatory factors early (2 weeks of DR). The inflammatory activation of *Egr2*^*−*^ M1 microglia may be earlier than that of *Egr2*^+^ M1 microglia. The upregulated inflammatory factors (*Il6*, *Tnf*, and *Ccl3*) in *Egr2*^*−*^ M1 microglia can bind to the corresponding receptors on the surface of *Egr2*^+^ M1 microglia, inducing inflammation, adhesion, and aggregation of *Egr2*^+^ M1 microglia [41–43]. However, all of these potential mechanisms remain at the theoretical stage, and the heterogeneity of microglia in disease and the transition from one response to another deserves to be confirmed by further studies.

### Activation mechanism of microglia

To characterize the mechanism of inflammation development in DR, we focused on analyzing the activation mode of *Egr2*^+^ M1 microglia and *Egr2*^*−*^ M1 microglia. Research has shown that overactivation of microglia is accompanied by morphological changes, migration, and accumulation around the ischemic area in the retina in the proliferative phase, which aggravates the original microvascular dysfunction [44].

**Fig. 3:**
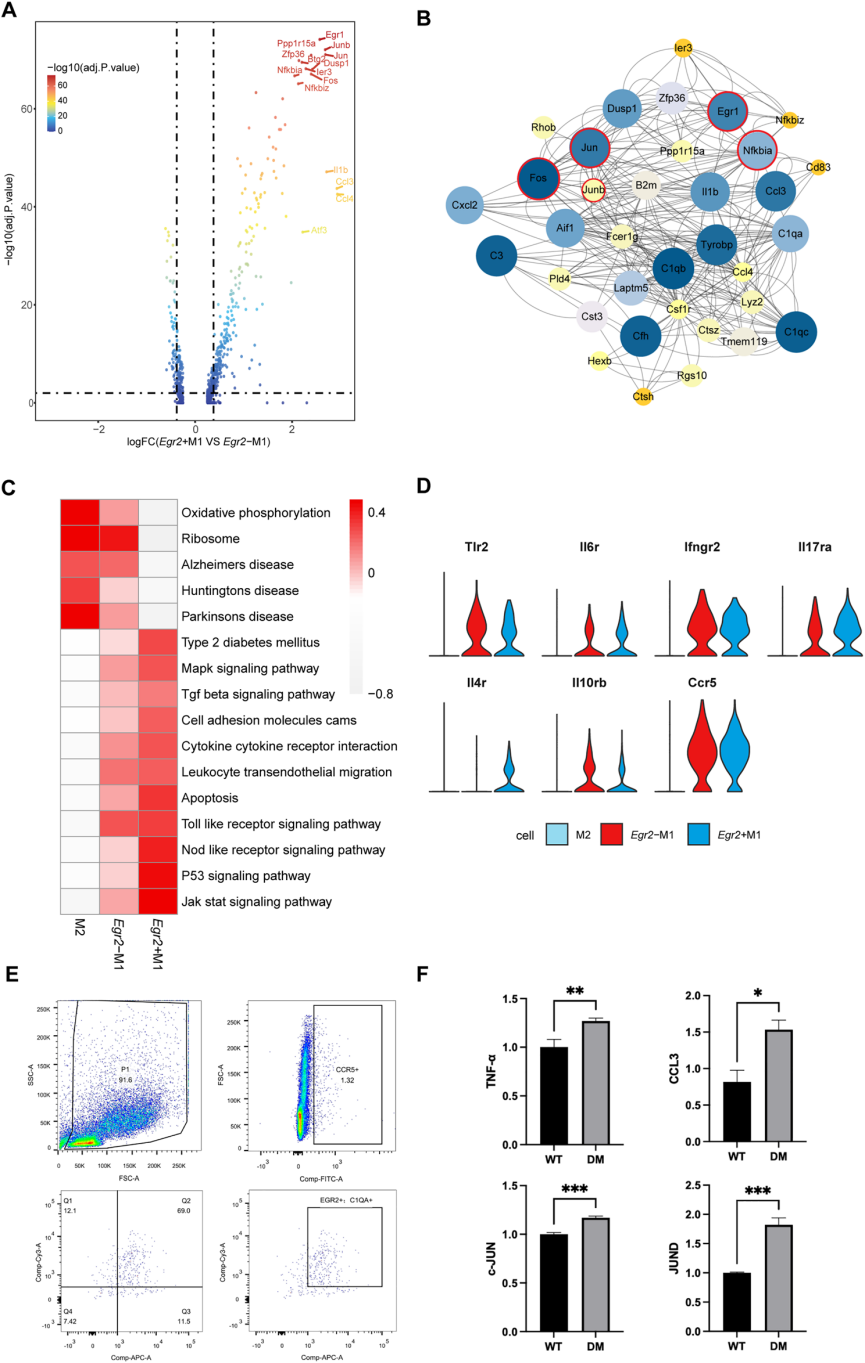
Activation mechanism of microglia. **A** Volcano plot showing the DEGs between *Egr2*^+^ M1 microglia and *Egr2*^*−*^ M1 microglia. The adjusted P value and gene expression (log fold change) were used for the plot. **B** PPI network showing the highly expressed proteins of *Egr2*^+^ M1 microglia. The size and color represent the combined score of each protein. Significant proteins are marked with red circles. **C** Heatmap showing the enrichment of pathways in different subtypes of microglia in GSVA. High expression is in red, and low expression is in blue. **D** Violin plot showing the expression of inflammatory receptors in the three groups of microglia. **E** Gating strategy for sorting *Egr2*^+^ M1 cells. *Egr2*^+^ M1 cells are gated as *Ccr5*^+^*C1qa*^+^*Egr2*^+^ cells. **F** The bar graph shows the qPCR results for *Egr2*^+^ M1 cells. * denotes a p-value of < 0.05, ** denotes a p-value of < 0.01 and *** denotes a p-value of < 0.001. The levels of inflammatory cytokines like Tnf and members of the AP-1 family were significantly elevated in *Egr2*^+^ M1 cells compared to normal cells

When comparing the DEGs of *Egr2*^+^ M1 microglia and *Egr2*^*−*^ M1 microglia, we noticed that the expression of three transcription factors, AP-1 (*Junb*, *Fos*, *Jun*), *Egr1*, and NF-κB (Fig. 3A), which have been indicated to promote the transcription of a variety of inflammatory factors [45, 46] and to have a synergistic effect [47], was upregulated in *Egr2*^+^ M1 microglia. We performed protein–protein interaction (PPI) network [48] analysis on *Egr2*^+^ M1 microglia, which confirmed their importance (Fig. 3B). Gene set variation analysis (GSVA) was performed on the three microglial subgroups, and we obtained pathways with significant difference [49] (Fig. 3C). M2 microglia exhibited upregulation of pathways associated with diseases such as Parkinson's disease, AD and Huntington's disease, which prompted us to focus on the similarities between microglia in the brain and microglia in the retina [50, 51]. M1 cells exhibited significant upregulation of inflammation-related pathways, confirming that they were in a highly inflammatory state. Since the expression of the MAPK [52] and JAK/STAT [53] pathways is consistent with the level of inflammation, inflammation is likely regulated by these two pathways.

In addition, we found that a variety of inflammatory factor receptors were coexpressed on M1 microglia. Most of them were activated to produce proinflammatory effects, while a few had the opposite effect (Fig. 3D). Some highly expressed receptors on M1 microglia (Toll-like receptors, *Il6* receptors, *Il17* receptors and Ifnγ receptors) (Fig. 3D) have previously been proven to significantly enhance inflammation [54–57]. However, targeted drugs that can inhibit one of these proinflammatory receptors cannot completely prevent the polarization of glial cells, which may be attributable to the coexpression of multiple receptors [58–60]. Anti-inflammatory receptors on microglia may also become key targets of clinical treatment. *Egr2*^*−*^ M1 microglia and *Egr2*^+^ M1 microglia both express the *Il10* receptor, and *Egr2*^+^ M1 microglia specifically expresses the *Il4* receptor (Fig. 3D). IL-4 and IL-10 signals also increase the polarization of M2 macrophages [58–60].

Studies have confirmed that activation of receptors for advanced glycation end products (RAGEs) in other diseases, such as liver ischemia/reperfusion injury and colon cancer, can stimulate the transcription of the *Egr1* and AP-1 families [61–64]. In early DR, RAGEs, NF-κB and inflammatory factors were simultaneously upregulated in *Egr2*^*−*^ M1 microglia. The activation of *Egr2*^*−*^ M1 microglia was also accompanied by upregulation of RAGEs, which bind to advanced glycosylation end products (AGEs), resulting in activation of NF-κB and promoting overexpression of proinflammatory mediators and RAGEs. Therefore, the RAGE/NF-κB signaling pathway may be of great significance to the activation of *Egr2*^*−*^ M1 microglia, but whether RAGEs are associated with AP-1 and *Egr1* deserves further investigation.

We sorted out *Egr2*^+^ M1 cells using flow cytometry and detected specific inflammatory factors and AP-1 family members using qPCR, which validated the properties of *Egr2*^+^ M1 (Fig. 3E, F). In conclusion, we identified two subgroups of M1 microglial activation-related receptors, transcription factors and pathways and revealed the possible mechanisms underlying their mutual regulation of activation. The activation of *Egr2*^*−*^ M1 microglia may be the driving factor of local inflammation in the retina: *Egr2*^*−*^ M1 microglia are activated and release proinflammatory factors in the early stage that bind to receptors on both *Egr2*^*−*^ M1 microglia and *Egr2*^+^ M1 microglia to enhance inflammation.

### Dynamics of the polarized phenotype of microglia

**Fig. 4:**
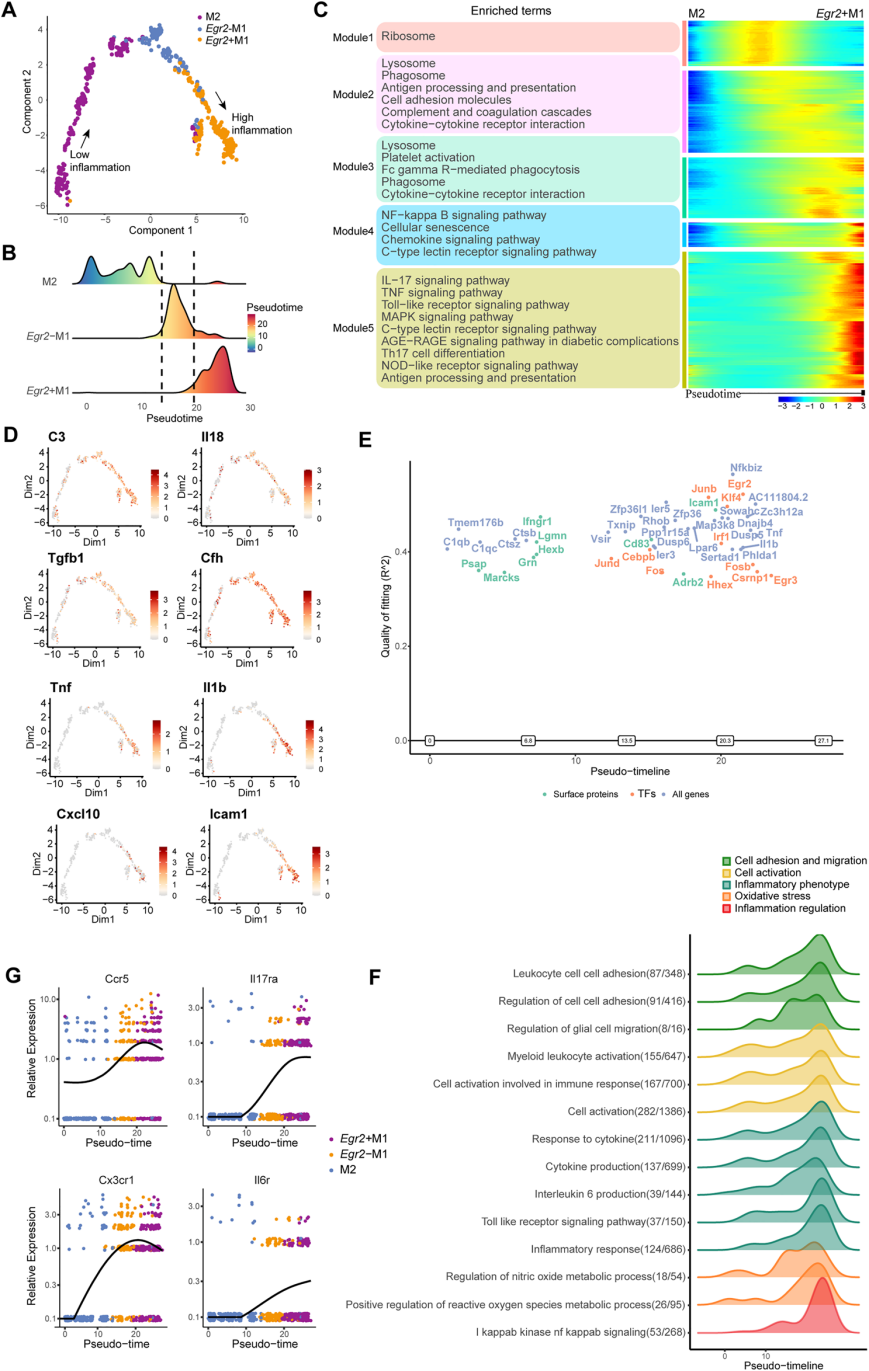
Dynamics of the polarized phenotype of microglia. **A** Pseudotime trajectory analysis showing the polarization characteristics of the three groups of microglia. Different colors represent different microglial subtypes. The arrows point to the start and end points of the trajectory. **B** Ridge plot showing the distribution of three groups of microglia in pseudotime. **C** Heatmap (right) showing changes in gene expression during pseudotime. Genes in the trajectory are divided into 5 modules. The KEGG analysis result (adjusted p < 0.05) corresponding to each module is on the left. **D** Feature plot showing the expression changes in selected genes over pseudotime. **E** Significant genes in the pseudotime analysis, including surface proteins, transcription factors (TFs), and all genes. **F** Ridge plot of the significant pathway (GO) terms over pseudotime. FDR < 0.05. **G** Gene expression of inflammatory receptors over pseudotime. The different colors correspond to different cell types

Studies have shown that hyperglycemia-induced M1/M2 polarization imbalance is closely related to DR [65]; thus, modulating the microglial polarization phenotype is a new therapeutic strategy. Since the inflammatory effect of *Egr2*^*−*^ M1 microglia is between that of M2 microglia and *Egr2*^+^ M1 microglia, we hypothesized that *Egr2*^*−*^ M1 microglia might be an M1/M2 transitional phenotype. To gain insight into the polarization dynamics of microglia, we used Monocle2 for pseudotime analysis of the three subtypes and visualized the trajectory in a t-distributed stochastic neighbor embedding (tSNE) plot (Fig. 4A and Additional file 1: Figure S4A). The two ends of the trajectory were M2 microglia and *Egr2*^+^ M1 microglia, and *Egr2*^*−*^ M1 microglia were in the middle of the track (Fig. 4B).

We identified the dynamically expressed genes along the trajectory and performed KEGG enrichment analysis. Most of the genes were related to inflammation, suggesting that this trajectory reflects the polarization phenotype of microglia. We divided these genes into 5 modules reflecting the dynamic changes in gene expression during the polarity change of microglia (Fig. 4C). The KEGG results also showed that the signaling pathways of inflammatory regulation, such as the MAPK and AGE-RAGE pathways (Fig. 4C), were significantly enriched at the end of the track. Combined with the trajectory, the results indicated that the polarity of microglia is continuous and that the intermediate *Egr2*^*−*^ M1 microglia may have bidirectional polarization ability.

Through a specific analysis of the characteristics of the 5 modules, we noticed that the genes of module 1 were mostly ribosomal genes and mainly expressed in M2 microglia, suggesting that the metabolism of M2 microglia was active. The genes of modules 2 and 3, including some inflammatory factors and complements, mainly existed in cells with transitional polar phenotypes. Inflammatory mediators and transcription factors were found in modules 4 and 5, which were mainly expressed in highly polar cells (*Egr2*^+^ M1 microglia). Under pathological conditions, the polar phenotype of the microglial population underwent changes: while some weakly inflammatory cells became moderately inflammatory ones, moderately inflammatory cells were also converted into highly inflammatory cells. In conclusion, microglia showed dynamic changes toward polarity enhancement under long-term hyperglycemia.

We observed that weakly inflammatory cells activated complement (*C3*, etc.) to complete the transition to moderately inflammatory cells. Moderately inflammatory cells mediated the release of many inflammatory factors, such as *Tnf* and *Il1β,* by upregulating transcription factors and then became highly inflammatory cells (Fig. 4D).

To discover the transcription factors and pathways that induce polar transformation, we used GeneSwitches to identify the most significant genes in the transformation trajectory (Fig. 4E). Through analysis of the pseudotime trajectory, we determined that the early stage was dominated by complement-related genes. Notably, transcription factors (AP-1 family [*Junb, Fos, Fosb*], *Egr2, Egr3, Hhex*, etc.) varied significantly along the trajectory, suggesting that they may act as switches to induce microglial polarization toward the M1 phenotype. This was also well reflected in the PPI analysis of module 5 (Additional file 1: Figure S4B). The GeneSwitches results showed pathway changes in the polarization trajectory (Fig. 4F). Among them, changes in the NF-κB signaling pathway may have been the keys to inducing the polarization of microglia to the proinflammatory type. Accordingly, we also observed that the receptors of some inflammatory cytokines were upregulated at different times along the trajectory, which may have played a role in inducing polarity (Fig. 4G).

### Role of macrophages in DR and the regulatory network of inflammatory cells

The recruitment and activation of macrophages are key factors in the occurrence and promotion of inflammation in retinal diseases [66, 67]. In proliferative DR, the increase in macrophages is closely related to leak into retinal capillaries [68]. In early DR, macrophages can also infiltrate and accumulate in the retina [69, 70], but the specific molecular mechanism of pathogenicity is unknown. We identified this rare cell population through single-cell technology and deeply studied how macrophages in early DR induce inflammation and how they cooperate with other cells to build a complex inflammatory regulatory network.

We compared the roles of macrophages and M1 microglia in mediating inflammation (Fig. 2F). Similar to M1 microglia, macrophages secrete inflammatory factors (*Il1β* and *Il12*) [71, 72] and express NAPDH oxidases involved in oxidative stress, such as *Rac2*, *Cybb* and *Ncf4* [73, 74]*.* However, the ability of macrophages to promote inflammation is weaker than that of M1 microglia. In addition, we observed that M1 microglia and macrophages secreted chemokines to mediate cell migration: *Egr2*^+^ M1 microglia mainly secreted *Ccl2* and *Cxcl2*, while macrophages mainly secreted *Ccl5* and *Ccl6*. Notably, the complement system that mediates early inflammation is not expressed in macrophages; thus, macrophages are not the primary cells mediating early inflammation [75].

**Fig. 5:**
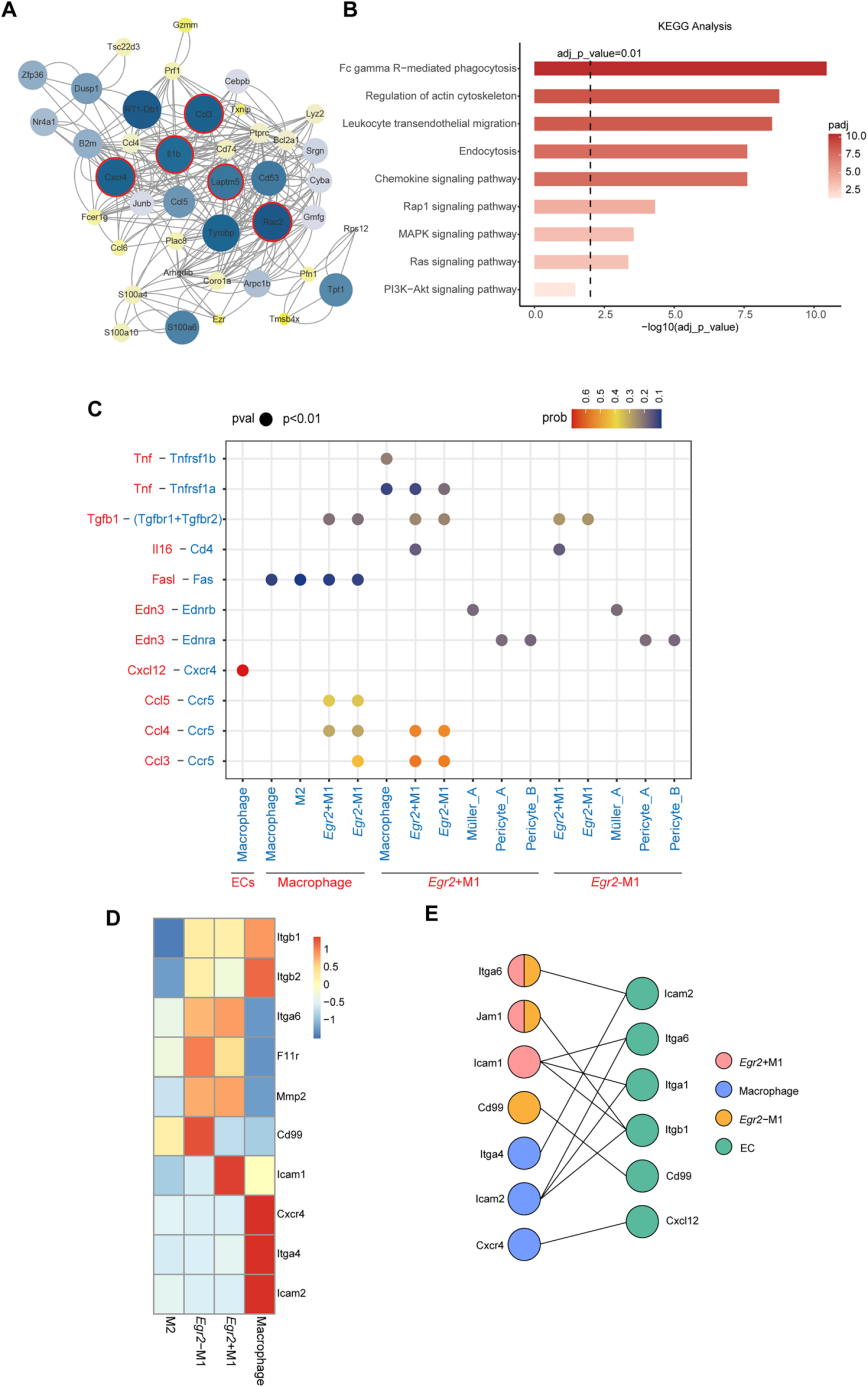
Role of macrophages in DR, inflammatory regulatory networks and adhesion mechanisms of inflammatory cells. **A** The PPI network shows the highly expressed proteins of macrophages (ribosomes removed). The size and color represent the combined score of each protein. Significant proteins are marked with red circles. **B** Bar plot showing the pathways from KEGG analysis. The color and length indicate the significance of pathway enrichment. **C** Bubble plot showing the important ligand–receptor pairs from CellChat analysis. The horizontal axis shows the communication relationship between cells. The longitudinal axis shows ligand–receptor pairs. The color indicates the communication probability, and the point size indicates the calculated p value. **D** Heatmap showing the average expression of differentially expressed adhesion molecules in microglia and macrophages. High expression is in red, and low expression is in blue. **E** Interactions between adhesion molecules and their receptors expressed by inflammatory cells and endothelial cells (ECs). Circles represent different types of adhesion molecules and their corresponding receptors. The colors represent the cell types: *Egr2*^+^ M1 microglia (pink), macrophages (blue), *Egr2*.^−^ M1 microglia (yellow), and ECs (green)

Then, we constructed a PPI network for the proteins highly expressed by macrophages (Fig. 5A). We found that these proteins (*Il1β*, *Ccl3*, *Cxcr4*, *Rac2*, and *Laptm5*) [71, 73, 76–78] were important in the protein interaction networks. KEGG analysis suggested that the MAPK, PI3K-Akt and Ras signaling pathways were upregulated in macrophages, which may be related to adhesion aggregation and chemokine release [52, 79, 80] (Fig. 5B).

The number of macrophages is low in early DR; however, macrophages can release inflammatory cytokines/chemokines. Hence, we focused on the cellular communication of inflammatory cells, including macrophages and microglia. We used CellChat to determine the specific relationship between cells in the inflammatory regulatory network (Fig. 5C). Macrophages, *Egr2*^+^ M1 microglia, and *Egr2*^−^ M1 microglia achieved mutual cell regulation through different inflammatory factors: *Egr2*^+^ M1 microglia acted on macrophages and *Egr2*^−^ M1 microglia (*Tnfrsf1b*, *Tnfrsf1a*) by releasing *Tnf-α*, *Egr2*^−^ M1 microglia acted on *Egr2*^+^ M1 microglia by releasing *Il16*, macrophages acted on M1 microglia by releasing *Ccl4* and *Ccl5*, and these inflammatory factors also acted on the cells that released them in an autocrine manner. In addition to playing roles in inflammation, these proinflammatory factors activated more inflammatory cells and induced the release of more inflammatory factors. We believe that macrophages, *Egr2*^+^ M1 microglia and *Egr2*^−^ M1 microglia induce complex inflammatory cascade reactions and enhance the damage of inflammation via inflammatory factors and their receptors. Macrophages can specifically express *FasL*, act on *Fas* on other cells, and rapidly mediate apoptosis by promoting the recruitment and activation of the apoptosis-initiating proteases *caspase-8* and *caspase-10* [81]*.* In addition, M1 microglia and Müller cells produce two subtypes of *TGFβ*, *TGFβ1* and *TGFβ2*, of which *TGFβ1* acts on M1 microglia themselves. The increased concentration of *TGF* in eyes is related to microvascular complications and tissue fibrosis in nonproliferative DR [82].

### Inflammatory cell adhesion damages the blood–retinal barrier (BRB)

Adhesion and aggregation of inflammatory cells lead to microvascular leakage in early DR by releasing inflammatory factors and disrupting the BRB [71, 83]. Our research also shows that inflammatory cells enhance damage to the BRB through adhesion and aggregation, release of matrix metalloproteases (MMPs) and enhancement of oxidative stress.

Here, we explored the adhesion factors on the surfaces of inflammatory cells and the corresponding receptors on endothelial cells (Fig. 5D, E). M2 microglia hardly expressed adhesion molecules. Integrin β (*Itgb1*, *Itgb2*) plays a key role in the adhesion of other inflammatory cells. M1 microglia can bind to the endothelial integrin receptor through Jam1 (*F11r*), and which *Egr2*^*−*^ M1 microglia specifically express *Cd99*, while *Egr2*^+^ M1 microglia specifically express *Icam1*. In early DR, endothelial cells affected by ischemia and hypoxia upregulate the expression of *Cxcl12* (the receptor of which is *Cxcr4* on macrophages) to recruit macrophages. The adhesion of macrophages can cause endothelial cell death [84, 85]. The different positions of microglia and macrophages relative to endothelial cells result in different adhesion patterns between them. Previous reports have mentioned that inflammation-induced oxidative stress leads to activation of ROS [86, 87], which can promote leukocyte adhesion and inflammation. We observed that both M1 microglia and macrophages upregulated NADPH oxidase, which may have strengthened oxidative stress.

It has been shown that elevated expression of MMPs in the retina may promote increased vascular permeability and disruption of the entire tight junction complex. MMP2 is thought to be critical for cellular integrity and cell survival, and diabetes activates MMP2 in the retina and its capillary cells, which can also increase cell membrane permeability and activate apoptosis in capillary cells by regulating mitochondrial function [88]. M1 cells highly express this protein, and whether they are involved in the regulation of retinal vascular permeability in the early stages of DR deserves further investigation.

## Discussion

Pericyte loss, basement membrane thickening and BRB damage are considered to be the earliest pathological changes in DR [89]. However, our previous work confirmed that the levels of *Tnf-α*, *Il1β* and other inflammatory factors are increased in the serum of 2-month-old rats, indicating that the intraretinal inflammatory response precedes the above microvascular changes and occurs throughout the entire process of DR [90]. Therefore, designing therapeutic targets for retinal inflammation will help improve the diagnosis and treatment of DR, making research on retinal inflammation particularly critical. We focused on early DR with single-cell sequencing analysis of 10 retinal cell types. Specifically, we focused on inflammatory regulatory networks centered on microglia and macrophages. Our work elucidates the possible cellular and molecular mechanisms responsible for early inflammation and microvascular leakage, providing new insights for early intervention in DR and broadening our horizons for the diagnosis of DR.

Previous studies have often divided microglia into two types: anti-inflammatory (M2) microglia and proinflammatory (M1) microglia [91]. We innovatively divided microglia into three subgroups (one type of M2 microglia and two types of M1 microglia) and deeply explored the inflammatory effects and activation sequences of the three subgroups. According to the specific expression of *Egr2*, the two M1-type microglial subtypes were named *Egr2*^+^ M1 microglia and *Egr2*^*−*^ M1 microglia. Immunofluorescence experiments confirmed the grouping results and clearly demonstrated the distribution characteristics of the three cell types in the retina. We explored the inflammatory effects of the three cell populations and found that M2 microglia were weakly inflammatory, similar to the findings in previous studies [92], and behaved as quiescent microglia. The newly differentiated *Egr2*^+^ M1 microglia were highly inflammatory, capable of releasing massive amounts of inflammatory factors, which are major causes of retinal inflammation. The *Egr2*^+^ M1 microglia exerted a strong proinflammatory effect during the whole process of DR, and these cells provide a new idea for inhibiting inflammation. The inflammatory nature of *Egr2*^*−*^ M1 microglia was between those of the two other subtypes. The *Egr2*^*−*^ M1 microglia secreted inflammatory mediators with weak proinflammatory effects, such as complement, playing a more important role in early DR; thus, these cells may become important targets for blocking the progression of DR. Our results indicate that there are subpopulations associated with different degrees of inflammation within the previously classified proinflammatory microglia. These findings provide more theoretical insights for understanding the heterogeneity among microglia and guiding precision therapy.

We noticed that highly inflammatory microglia exhibited significant upregulation of three types of transcription factors, AP-1, *Egr1*, and NF-κB. In addition, We identified the critical roles of the NF-κB, MAPK and JAK/STAT pathways in the inflammation of early DR. Inflammatory factor receptors on microglia not only characterize the inflammation of microglia but also participate in the activation of microglia. Activation of microglia can transmit signals through inflammatory receptors and activate the downstream MAPK and JAK/STAT pathways, and the activated pathways stimulate the transcriptional regulation of inflammatory factors via transcription factors. A similar situation can be found in the retinal microglia of cynomolgus monkeys with diabetes [93]. Researchers have found that the activation of microglia is closely related to inflammatory factors like TNF and NF-κB pathway. These results further elucidate the importance of microglia in DR. By deeply exploring the activation mechanisms of microglia, we have provided a theoretical therapeutic target for clinical inhibition of inflammation in the early stage of DR.

The activation of microglia in the retinas of diabetic rats is closely related to the inflammatory response. Upon activation, microglia change their morphology from branched to amoeba-like, release inflammatory factors in large quantities, and migrate to the outer retina [10]. The exact mechanism of microglial activation in DR is not fully understood, and the timing of microglial activation during the course of DR is difficult to determine. Activation of both subtypes of M1-type microglia can characterize the inflammatory effect of the retina; however, we found that the order of activation of the two subtypes was not consistent. *Egr2*^*−*^ M1 microglia were activated earlier, showing activation at 2 weeks after diabetes modeling. However, the inflammation of *Egr2*^+^ M1 microglia was attenuated in the early stage of modeling and was upregulated at 4 weeks, indicating that the activation time of these microglia was later than that of *Egr2*^*−*^ M1 microglia. Our study found that RAGE and NF-κB expression was upregulated in *Egr2*^*−*^ M1 microglia in the early stage. These findings indicate that RAGEs activate the NF-κB signaling pathway to mediate inflammation and oxidative stress, which is consistent with previous studies [94], suggesting that the RAGE-NF-κB axis may be involved in *Egr2*^*−*^ M1 microglial activation. We believe that the inflammatory factors released by *Egr2*^*−*^ M1 microglia after activation can act on Egr2^+^ M1 microglia, which may be an important link in the amplification of the inflammatory cascade. Therefore, early blockade of the RAGE/NF-κB axis of *Egr2*^*−*^ M1 microglia has broad therapeutic prospects.

Switching the phenotype of microglia is a new therapeutic strategy that can inhibit inflammation and exert neuroprotective effects mainly by promoting the microglial polarization in the M2 direction. Regulating the polarity of microglia is a promising therapeutic direction for promoting central and retinal neuronal damage repair. In many neuroinflammatory diseases, such as AD [95] and stroke [96], the activation state of microglia is highly consistent with the manifestation of the disease. However, the microglial activation mechanism and the specific polarity regulation-inducing signals remain to be further elucidated. We probed the polarization dynamics of microglia and obtained a polarization trajectory from M2 microglia to *Egr2*^*−*^ M1 microglia to *Egr2*^+^ M1 microglia. This trajectory suggested that microglia would exhibit enhanced polarity under long-term hyperglycemia. We analyzed the molecular mechanism of the polarization transition with Monocle2 and GeneSwitches and found that the AP-1 family of transcription factors and the NF-κB signaling pathway can induce the transition of microglia in a hyperinflammatory direction. Our study provides further insights into balancing the polarization between the M1 and M2 subtypes to promote the repair of optic neuron damage in early DR and provides a therapeutic strategy for neuroprotection in CNS diseases.

Studies have shown that in healthy conditions, white blood cells temporarily stagnate in retinal vessels. In late DR, the cells stagnate for longer or stasis occurs, and this is accompanied by capillary occlusion, retinal ischemia and hypoxia [97]. In early DR, the main part of the inflammatory response is the retinal innate immune cell–microglia response; few macrophages can be detected. Therefore, the effects of macrophages in early DR have remained unclear. We took advantage of single-cell sequencing to deeply explore the mechanisms of macrophages in early DR. Macrophages mainly exerted a proinflammatory effect, secreted proinflammatory factors and participated in the release of oxidative stress products. Macrophages had a weaker inflammatory effect than *Egr2*^+^ M1 microglia and weakly expressed complement. However, macrophages released chemokines, which mediate the adhesion and aggregation of other inflammatory cells.

Adhesion of inflammatory cells is an important cause of retinal microvascular occlusion, and elevated levels of soluble adhesion molecules correlate with the severity of DR [98]. We analyzed the mechanism of adhesion to the vascular endothelium for M1 microglia and macrophages and found that these cell types expressed different adhesion molecules. Among them, *Egr2*^*−*^ M1 microglia specifically expressed *Cd99*, *Egr2*^+^ M1 microglia specifically expressed *Icam1*, and macrophages specifically expressed *Icam2*, *Itga4*, and *Cxcr4*, which provides us with a new idea to inhibit the adhesion of inflammatory cells.

Notably, we investigated the communication networks between cells and detected complex, dynamic interactions between inflammatory cells and other cells in the retina throughout the development of DR. In addition to interacting with each other, inflammatory cells can regulate neuronal cells and endothelial cells. Other cells, mainly endothelial cells, are also involved in the regulation of inflammatory cells. Therefore, in-depth study of the inflammatory regulatory network may help to develop a new strategy to prevent retinal damage in early DR.

In conclusion, we constructed an inflammatory regulatory network centered on microglia by constructing a retinal transcriptional atlas in early DR. We defined a new classification standard of microglia and studied the activation characteristics of different subtypes of cells, providing a theoretical basis for the diagnosis of early DR. We also explored the molecular mechanisms mediating early chronic inflammation in DR and provide an important therapeutic target for early clinical intervention in the progression of DR. Our study characterizes the polarization dynamics of microglia in DR and contributes ideas for the treatment of neuroinflammatory diseases through modulation of M1/M2 polarity. Furthermore, we investigated macrophages, which are less abundant than other cell types in DR, and our findings provide insights into the mechanisms of adhesion of macrophages and microglia to endothelial cells.

Through this cell atlas, we found that microglia show the most obvious differential expression changes in early DR and reveal the changes in microglia in a high-glucose microenvironment at the single-cell level. Thus, precise detection of these target cells can shorten the amount of time needed for drug screening and accelerate clinical application. Our comprehensive analysis will help achieve early reversal and control the occurrence and progression of DR.

## Conclusions

Through Single-cell sequencing technology, we found unique microglia subtypes and analyzed the polarization characteristics of cells at the early stage of DR and the relationship with other cells. However, due to the small sample size, more validation experiments need to be further implemented to clarify the correctness of our conclusions.

### Supplementary Information

**Additional file 1: Figure S1. (A)** The figure shows the number of high-quality cells retained for each sample after quality control. **(B)** UMAP plot shows 33 clusters (Red Blood Cells excluded). **(C)** Featureplot shows the classic marker genes of each cell type. Color scale: red, high expression; gray, low expression. **(D)** Pie graph and histogram show the number of each cell type. **(E)** Bar plot shows the number of each cell type in different stages of DR. **(F)** Gene-expression heatmap of the top marker genes for each cell type. Color scale: red, high expression; gray, low expression. **Figure S2. (A)** Histogram shows the GO analysis results of Rods. Color and length indicate the adjust p-values of each pathway. **(B)** Featureplot shows the expression of *Opn1mw* and *Opn1sw* in Cones. Color scale: red and oringe, high expression; gray, low expression. **(C)** Venn plot shows the number of cells expressing *Opn1mw* and *Opn1sw* genes in Cones. The intersection area represents the number of *Opn1mw* + *Opn1sw* + cells, and the blank area represents the number of *Opn1mw-Opn1sw-* cells. **(D)** Violin plot shows the expression of marker genes in five BC subgroups. **(E)** Bubble plot shows the markers of AC and AC/HC. The size of each circle is proportional to the percentage of cells expressing the gene. The color of each circle represents the level of gene expression in the cell. **(F)** Ridge plot shows the expression of marker genes in AC/HC. Onecut1 is the marker for HC. *Slc6a9* and *Gjd2* are markers for AC. **(G)** Ridge plots shows the expression of markers of microglia (*C1qa*, *Aif1*, *Apoe*, *Tmem119*), blood derived macrophages (*Cxcr4*, *Cd53*, *Ptprc*) and perivascular macrophages (*Cd163*, Mrc1) in macrophages. **(H)** The outgoing and incoming communication pathways associated with inflammation in non-neuronal cells. Color scale: green, high relative strength; white, low relative strength. **Figure S3. (A)** UMAP plot of three subtypes of microglia (left) and a magnified version (right). **(B)** Heatmap shows the highly expressed genes of three subtypes of microglia. Color scale: red, high expression; gray, low expression. **(C)** Featureplot shows the expression of marker genes in microglia. *C1qa* is used to identify microglia, *Tmem119* and *Ccr5* are used to distinguish M1 and M2, and *Egr2* is used to distinguish two subgroups of M1. **(D)** Immunofluorescent labelling for *C1qa*(red), *Ccr5*(green), and DAPI nuclear staining (blue) in the rat retina. M1 is indicated by arrows and M2 is indicated by arrowheads (first row, 20X). Immunofluorescent labelling for *Ccr5*(red), *Egr2*(green), and DAPI nuclear staining (blue) in the rat retina. *Egr2* + M1 is indicated by arrows and *Egr2-*M1 is indicated by arrowheads (second row, 20X). Scale bar 50 um. **(E)** Immunofluorescent labelling for *C1qa*(red), *Ccr5*(green), and DAPI nuclear staining (blue) in the rat retina. M1 is indicated by arrows and M2 is indicated by arrowheads (left, 20X; first row, 40X). Immunofluorescent labelling for *Ccr5*(red), *Egr2-*(green), and DAPI nuclear staining (blue) in the rat retina. *Egr2* + M1 is indicated by arrows and *Egr2-*M1 is indicated by arrowheads (second row, 40X). Scale bar 50 um(left); 20um(right). **(F)** Immunofluorescent labelling for *C1qa*(red), *Ccr5*(green), and DAPI nuclear staining (blue) in the rat retina. M1 is indicated by arrows and M2 is indicated by arrowheads (63X). Scale bar 20 um. **Figure S4. (A)** Pseudotime trajectory analysis shows the polarization characteristics of three groups of microglia. Different colors represent different microglia subtypes. The arrows point to the start and end points of the trajectory. **(B)** The histogram shows the PPI analysis results of TOP 30 genes in Module 5, and the length represents the number of nodes. **Table S1.** Markers of major cell types. **Table S2.** New markers for each major cell population. **Table S3.** Blood glucose levels and weight.

## Data Availability

scRNAseq data were submitted to the National Center for Biotechnology Information Gene Expression Omnibus database under accession numbers GSE209872. Samples from GSE168908, GSM6205479, GSM7529034, GSM7273565 and GSM5769880 were also downloaded to be used in our research.
