# Supplementary material for: Single-cell RNA sequencing reveals roles of unique retinal microglia types in early diabetic retinopathy
Source: Diabetol Metab Syndr. 2024 Feb 26;16:49. doi: 10.1186/s13098-024-01282-3 (PMC10895757; doi:10.1186/s13098-024-01282-3)
Supplement: Supplementary file 1 — Additional file 1: Figure S1. (A) The figure shows the number of high-quality cells retained for each sample after quality control. (B) UMAP plot shows 33 clusters (Red Blood Cells excluded). (C) Featureplot shows the classic marker genes of each cell type. Color scale: red, high expression; gray, low expression. (D) Pie graph and histogram show the number of each cell type. (E) Bar plot shows the number of each cell type in different stages of DR. (F) Gene-expression heatmap of the top marker genes for each cell type. Color scale: red, high expression; gray, low expression. Figure S2. (A) Histogram shows the GO analysis results of Rods. Color and length indicate the adjust p-values of each pathway. (B) Featureplot shows the expression of Opn1mw and Opn1sw in Cones. Color scale: red and oringe, high expression; gray, low expression. (C) Venn plot shows the number of cells expressing Opn1mw and Opn1sw genes in Cones. The intersection area represents the number of Opn1mw + Opn1sw + cells, and the blank area represents the number of Opn1mw-Opn1sw- cells. (D) Violin plot shows the expression of marker genes in five BC subgroups. (E) Bubble plot shows the markers of AC and AC/HC. The size of each circle is proportional to the percentage of cells expressing the gene. The color of each circle represents the level of gene expression in the cell. (F) Ridge plot shows the expression of marker genes in AC/HC. Onecut1 is the marker for HC. Slc6a9 and Gjd2 are markers for AC. (G) Ridge plots shows the expression of markers of microglia (C1qa, Aif1, Apoe, Tmem119), blood derived macrophages (Cxcr4, Cd53, Ptprc) and perivascular macrophages (Cd163, Mrc1) in macrophages. (H) The outgoing and incoming communication pathways associated with inflammation in non-neuronal cells. Color scale: green, high relative strength; white, low relative strength. Figure S3. (A) UMAP plot of three subtypes of microglia (left) and a magnified version (right). (B) Heatmap shows the highly expressed ge [file 13098_2024_1282_MOESM1_ESM.pdf]

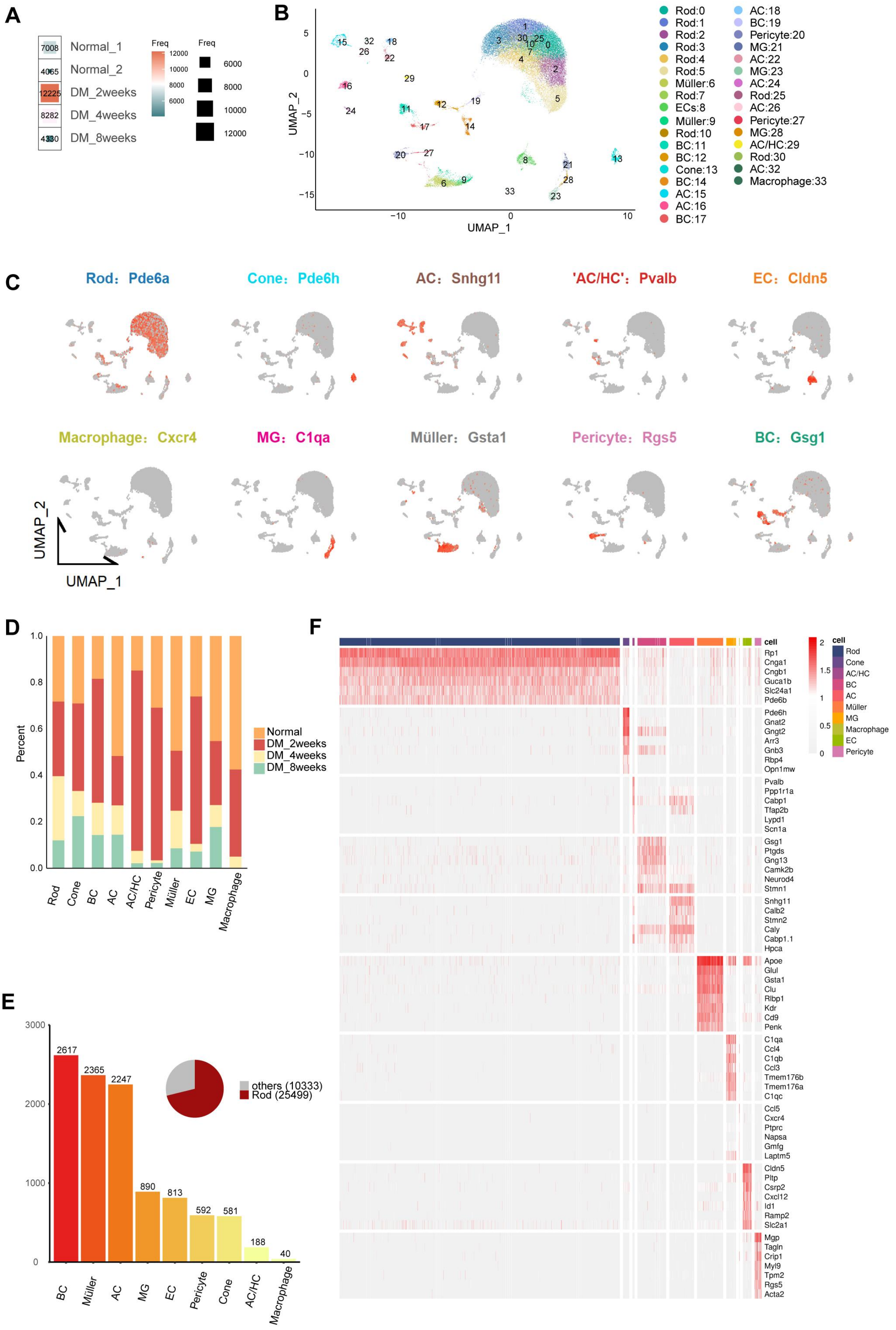

Figure S1. **(A)** The figure shows the number of high-quality cells retained for each sample after quality control. **(B)** UMAP plot shows 33 clusters (Red Blood Cells excluded). **(C)** Featureplot shows the classic marker genes of each cell type. Color scale: red, high expression; gray, low expression. **(D)** Pie graph and histogram show

the number of each cell type. **(E)** Bar plot shows the number of each cell type in different stages of DR. **(F)** Gene-expression heatmap of the top marker genes for each cell type. Color scale: red, high expression; gray, low expression.

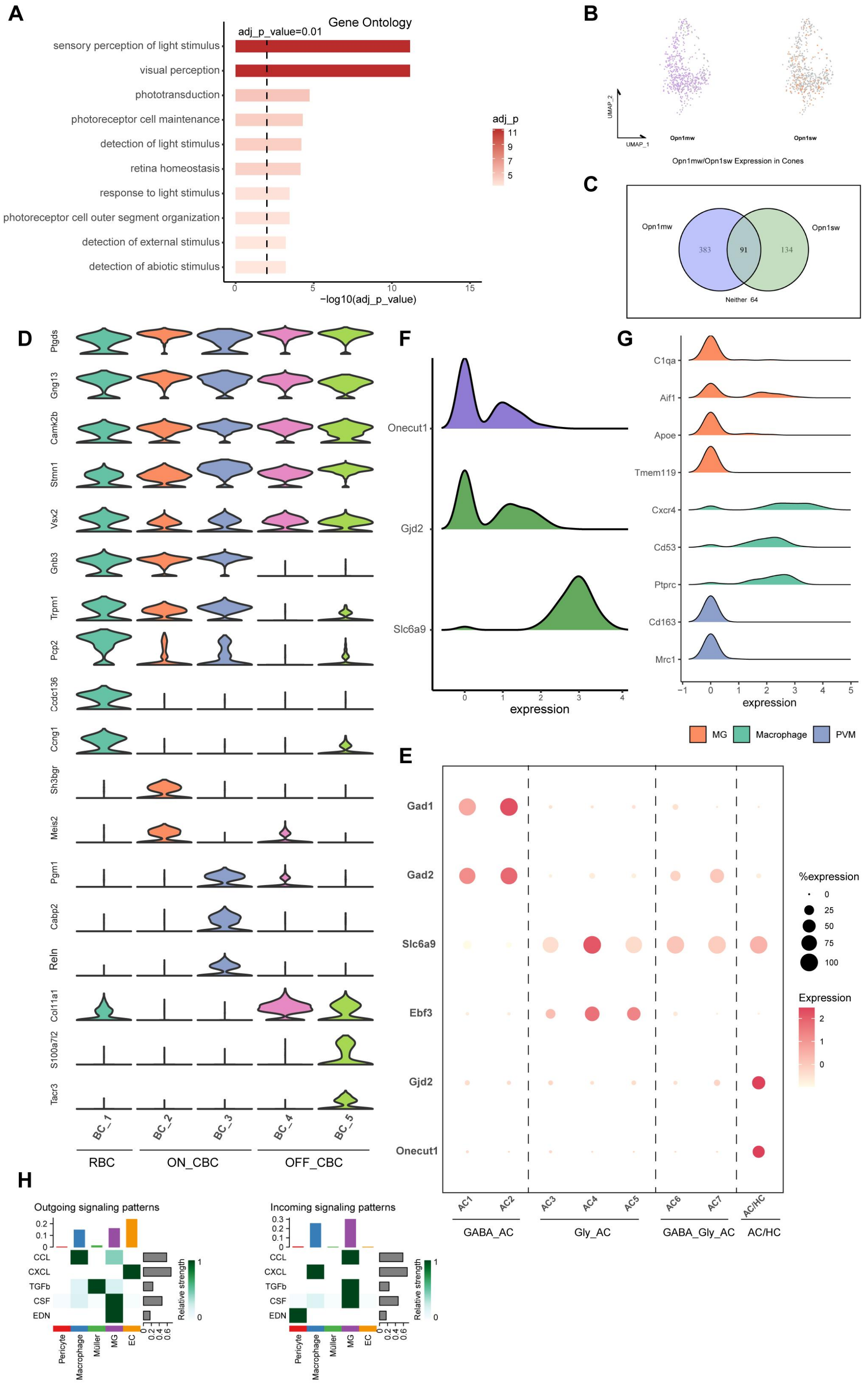

Figure S2. **(A)** Histogram shows the GO analysis results of Rods. Color and length indicate the adjust p-values of each pathway. **(B)** Featureplot shows the expression of *Opn1mw* and *Opn1sw* in Cones. Color scale: red and oringe, high expression; gray, low expression. **(C)** Venn plot shows the number of cells expressing *Opn1mw* and *Opn1sw* genes in Cones. The intersection area represents the number of $Opn1mw^{+}Opn1sw^{+}$ cells, and the blank area represents the number of $Opn1mw^{-}Opn1sw^{-}$ cells. **(D)** Violin plot shows the expression of marker genes in five BC subgroups. **(E)** Bubble plot shows the markers of AC and AC/HC. The size of each circle is proportional to the percentage of cells expressing the gene. The color of each circle represents the level of gene expression in the cell. **(F)** Ridge plot shows the expression of marker genes in AC/HC. Onecut1 is the marker for HC. *Slc6a9* and *Gjd2* are markers for AC. **(G)** Ridge plots shows the expression of markers of microglia (*C1qa*, *Aif1*, *Apoe*, *Tmem119*), blood derived macrophages (*Cxcr4*, *Cd53*, *Ptprc*) and perivascular macrophages (*Cd163*, Mrc1) in macrophages. **(H)** The outgoing and incoming communication pathways associated with inflammation in non-neuronal cells. Color scale: green, high relative strength; white, low relative strength.

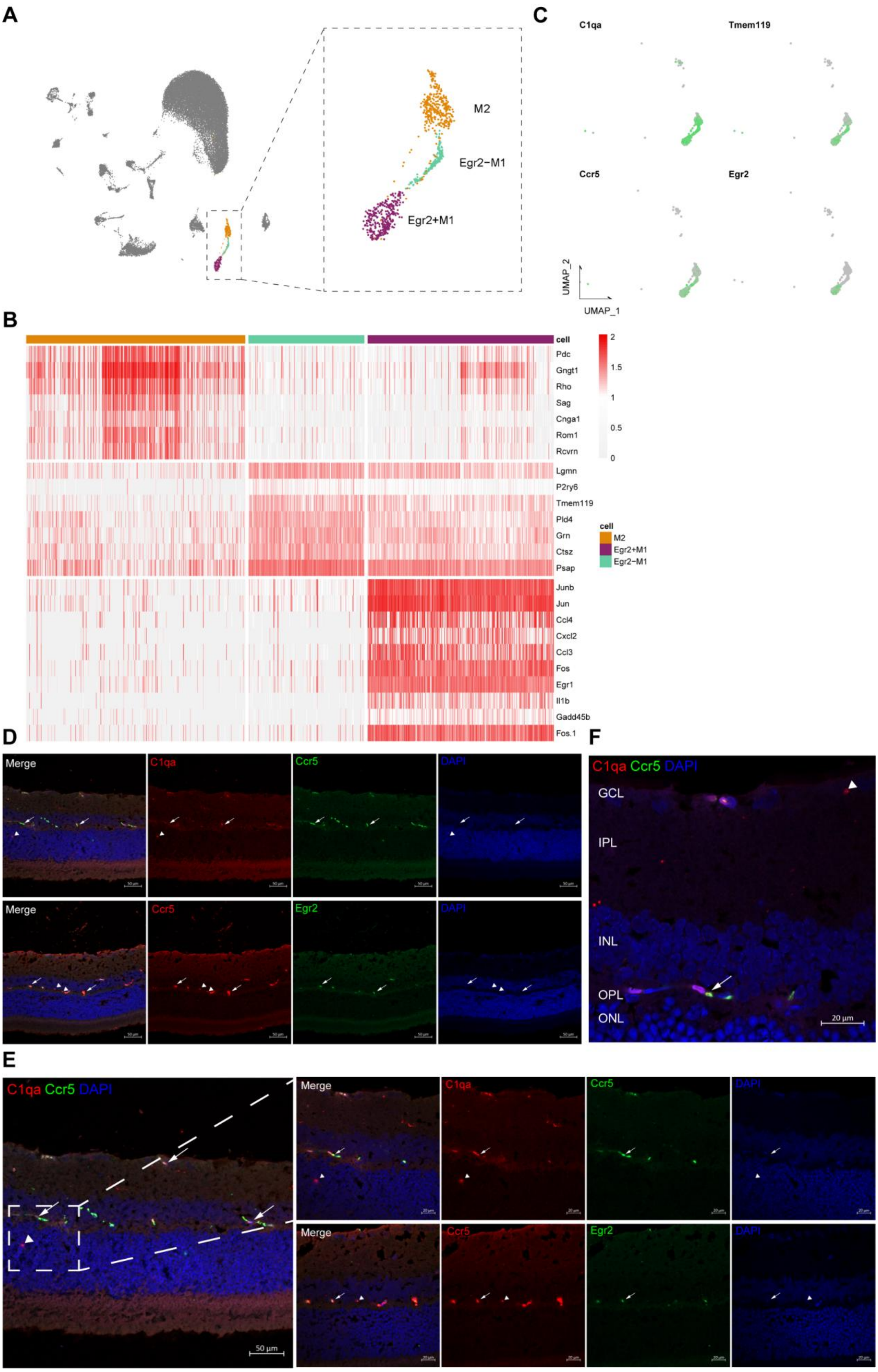

Figure S3. (A) UMAP plot of three subtypes of microglia (left) and a magnified version (right). **(B)** Heatmap shows the highly expressed genes of three subtypes of

microglia. Color scale: red, high expression; gray, low expression. **(C)** Featureplot shows the expression of marker genes in microglia. *C1qa* is used to identify microglia, *Tmem119* and *Ccr5* are used to distinguish M1 and M2, and *Egr2* is used to distinguish two subgroups of M1. **(D)** Immunofluorescent labelling for *C1qa*(red), *Ccr5*(green), and DAPI nuclear staining (blue) in the rat retina. M1 is indicated by arrows and M2 is indicated by arrowheads (first row, 20X). Immunofluorescent labelling for *Ccr5*(red), *Egr2*(green), and DAPI nuclear staining (blue) in the rat retina. *Egr2*$^{+}$M1 is indicated by arrows and *Egr2*$^{-}$M1 is indicated by arrowheads (second row, 20X). Scale bar 50 um. **(E)** Immunofluorescent labelling for *C1qa*(red), *Ccr5*(green), and DAPI nuclear staining (blue) in the rat retina. M1 is indicated by arrows and M2 is indicated by arrowheads (left, 20X; first row, 40X). Immunofluorescent labelling for *Ccr5*(red), *Egr2*$^{-}$(green), and DAPI nuclear staining (blue) in the rat retina. *Egr2*$^{+}$M1 is indicated by arrows and *Egr2*$^{-}$M1 is indicated by arrowheads (second row, 40X). Scale bar 50 um(left); 20um(right). **(F)** Immunofluorescent labelling for *C1qa*(red), *Ccr5*(green), and DAPI nuclear staining (blue) in the rat retina. M1 is indicated by arrows and M2 is indicated by arrowheads (63X). Scale bar 20 um.

A

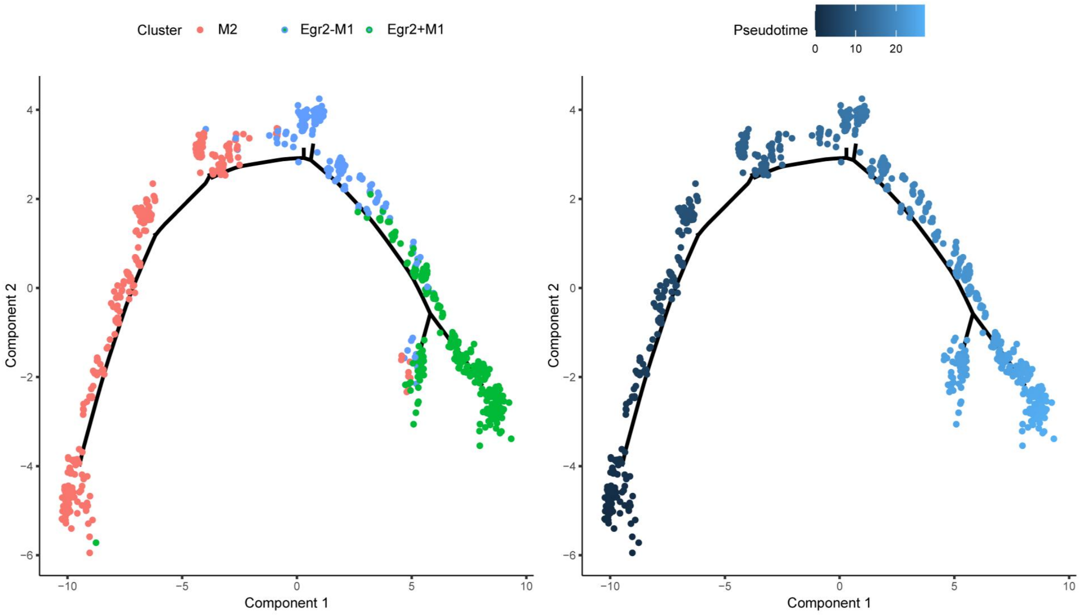

B

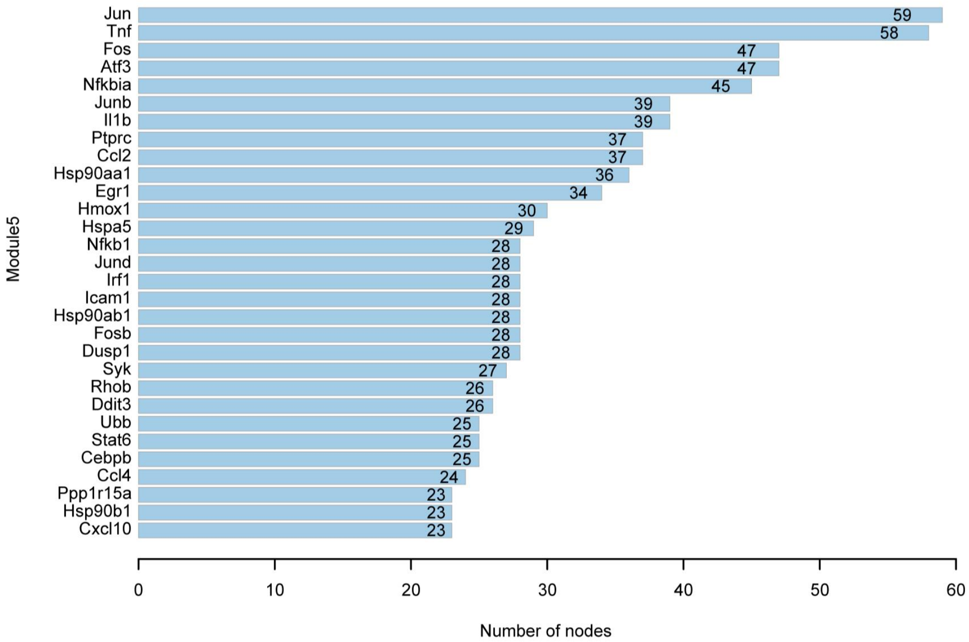

Figure S4. (A) Pseudotime trajectory analysis shows the polarization characteristics of three groups of microglia. Different colors represent different microglia subtypes. The arrows point to the start and end points of the trajectory. **(B)** The histogram shows the PPI analysis results of TOP 30 genes in Module 5, and the length represents the number of nodes.

Supplementary Table 1　 Markers of major cell types

**Cell Types**	**Markers**
Rod	*Pde6a, Rho, Sag, Gnat1, Nrl*
S Cone<br>M/L Cone	*Pde6h, Arr3, Gnat2, Opn1sw, Ccdc136, Ttr*<br>*Pde6h, Arr3, Gnat2, Opn1mw/Opn1lw, Vopp1, Lmo4*
HC	*Pvalb, Lhx1, Pax6, Onecut1*
GABA_AC<br>SEG AC<br>A-II AC	*Snhg11, Pax6, Slc32a1, Crabp1, Nrxn2, Gad1/Gad2, Maf, Tfap2a*<br>*Snhg11, Pax6, Slc32a1, Crabp1, Nrxn2, Slc6a9, Ebf3*<br>*Snhg11, Pax6, Slc32a1, Crabp1, Nrxn2, Slc6a9, Gjd2*
RBC<br>ON CBC<br>OFF CBC	*Gsg1, Pax6, Slc6a9, Vsx2, Otx2, Prkca, lsl1, Grm6, Cabp5, Vstm2b, Casp7, Rpa1*<br>*Gsg1, Pax6, Slc6a9, Vsx2, Otx2, App, Scgn, Vsx1, Isl1, Grm6*<br>*Gsg1, Pax6, Slc6a9, Vsx2, Otx2, App, Scgn, Vsx1, Grin2b, Grik1*
Müller	*Gsta1, Pax6, Rlbp1, Aqp4, Slc1a3, Apoe, Dkk3, Gpr37, Rax, Hes1, Notch1, Glul*
Microglia	*C1qa, Tmem119, Cx3cr1, Apoe, Pax6, P2ry12, Aif1*
EC	*Vwf, Pecam1, Cldn5, Cdh5, Tek, Kdr, Flt1*
Pericyte	*Rgs5, Kcnj8, Myl9, Cspg4, Pdgfrb, Myh11, Acta2*
Macrophage	*Cxcr4, Cd53, Ptprc*

Supplementary Table 2　New markers for each major cell population

Cell types	Clusters	Classic markers	New markers
Rod	0，1，2，3，4，5，7，10，25，30	*Pde6a, Rho, Sag*	*Rp1, Cnga1, Cngb1, Guca1b, Slc24a1, Pde6b*
Cone	13	*Pde6h, Arr3, Opn1sw, Opn1mw*	*Gngt2, Gnb3*
AC/HC	29	*Slc6a9, Gjd2, Onecut1, Pvalb*	*Ppp1r1a, Tfap2b, Lypd1, Scn1a, Ndrg4*
BC_1<br>BC_2<br>BC_3<br>BC_4<br>BC_5	19 (RBC)<br>12 (ON CBC)<br>14 (ON CBC)<br>11 (OFF CBC)<br>17 (OFF CBC)	*Gsg1, Vsx2, Prkca*<br>*Gsg1, Vsx2, App, Isl1, Grm6*<br>*Gsg1, Vsx2, App, Isl1, Grm6*<br>*Gsg1, Vsx2, App, Grin2b, Grik1*<br>*Gsg1, Vsx2, App, Grin2b, Grik1*	*Gng13, Ptgds, Ccdc136, Ccngl, Pcp2*<br>*Gng13, Ptgds, Meis2, Sh3bgr*<br>*Gng13, Ptgds, Pgm1, Cabp2*<br>*Gng13, Ptgds, Col11a1*<br>*Gng13, Ptgds, S100a7l2, Col11a1, Tacr3*
GABA_AC<br>Gly_AC<br>Gly_SEG AC<br>GABA_Gly_AC	15, 24<br>18<br>16, 22<br>26, 32	*Snhg11, Pax6, Slc32a1, Gad1, Gad2*<br>*Snhg11, Pax6, Slc32a1, Slc6a9*<br>*Snhg11, Pax6, Slc32a1, Slc6a9, Ebf3*<br>*Snhg11, Pax6, Slc32a1, Slc6a9, Gad2*	*Caly, Stmn2, Ly6h*<br>*Caly, Stmn2, Ly6h*<br>*Caly, Stmn2, Ly6h*<br>*Caly, Stmn2, Ly6h*
Müller	6, 9	*Apoe, Glul, Gpr37, Rlbp1, Gsta1*	*Kdr, Cd9, Penk*
M2<br>Egr2+M1<br>Egr2-M1	21 (microglia)<br>23 (microglia)<br>28 (microglia)	*Aif1, C1qa*<br>*Aif1, C1qa, Tmem119, P2ry12, Cx3cr1*<br>*Aif1, C1qa, Tmem119, P2ry12, Cx3cr1*	*Tmem176b,Tmem176a,Anxa3,Pdc*<br>*Tmem176b,Tmem176a,Anxa3,Ccr5,Egr2*<br>*Tmem176b,Tmem176a,Anxa3,Ccr5*
EC	8	*Ocln, Cldn5*	*Pltp, Csrp2, Cxcl12, Id1, Ramp2, Slc2a1*
Pericyte	20, 27	*Cspg4, Pdgfrb, Acta2, Rgs5*	*Mgp, Crip1, Tpm2*
Macrophage	33	*Cxcr4, Cd53, Ptprc*	*B2m, Coro1a, Arpc1b, Srgn, Gmfg, Laptm5*

Supplementary Table 3 Blood glucose levels and weight

	15th, November		18th, November	
	Weight (g)	Blood sugar(mmol/L)	Weight (g)	Blood sugar(mmol/L)
Experience group	542	6.6	495	26.2
	580	7.6	518	22.2
	523	6.1	447	24.2
Control group	507	5.8	509	6.6
	559	6.1	552	6.3
	534	6.3	542	6.2
	22th, November		29th, November	
	Weight (g)	Blood sugar(mmol/L)	Weight (g)	Blood sugar(mmol/L)
Experience group	467	H1	420	H1
	495	26.4	481	H1
	443	30.5	446	H1
Control group	521	5.6	532	7.4
	581	6	584	7
	563	5.9	587	7.5
	5th, December		12th, December	
	Weight (g)	Blood sugar(mmol/L)	Weight (g)	Blood sugar(mmol/L)
Experience group				
	512	H1	530	H1
	483	H1	471	H1
Control group	562	6.9	588	10.3
	619	6.7	654	9.7
	607	5.9	640	8.6
	26th, December		9th, January	
	Weight (g)	Blood sugar(mmol/L)	Weight (g)	Blood sugar(mmol/L)
Experience group				
	486	H1	465	H1
Control group	602	9	623	10.1
	678	6.6	684	6.8
	656	7.9	671	8.3
H1 indicates that the blood glucose instrument is out of range				
